# Barriers to pregnancy and parenthood during urology residency across Europe

**DOI:** 10.1002/bco2.70181

**Published:** 2026-03-31

**Authors:** Luca Afferi, Gaelle Margue, Carlos Toribio‐Vázquez, Belle M. Melsert, Loic Baekelandt, Anna Goujon, Mehmet Ozalevli, Enrico Checcucci, Anna Gugerli Lazos, Carolin Siech, Juan Luis Vásquez, Laura S. Mertens, Evangelos Liatsikos, Bhaskar K. Somani, Benjamin Pradere, Jessica Whitburn, Stephen Wyler, Alberto Breda, Veronique Phé, Joel S. Weissman, Erika Rangel, the European Society of Residents in Urology (ESRU)

**Affiliations:** ^1^ Department of Urology Kantonsspital Aarau Aarau Switzerland; ^2^ Harvard T.H. Chan School of Public Health Boston Massachusetts USA; ^3^ Department of Surgery and Morphological Science Universitat Autonoma de Barcelona Barcelona Spain; ^4^ Department of Urology Fundació Puigvert Barcelona Spain; ^5^ Service d'urologie, Department of Urology Bordeaux University Hospital, CHU de Bordeaux Bordeaux France; ^6^ HLA Moncloa University Hospital Madrid Spain; ^7^ Department of Surgical Oncology (Urology) Netherlands Cancer Institute, Antoni van Leeuwenhoek Hospital Amsterdam The Netherlands; ^8^ Department of Urology University Hospitals Leuven Leuven Belgium; ^9^ Department of Urology Rennes University Hospital Rennes France; ^10^ Department of Nephrology Rennes University Hospital Rennes France; ^11^ Gaziosmanpaşa Training and Research Hospital Istanbul Turkey; ^12^ Department of Urology Gaziosmanpaşa Training and Research Hospital, University of Health Sciences Istanbul Turkey; ^13^ Department of Surgery FPO‐IRCCS Candiolo Cancer Institute Candiolo Italy; ^14^ Department of Urology Universitätsspital Zürich Zürich Switzerland; ^15^ Department of Urology Goethe University Frankfurt, University Hospital Frankfurt am Main Germany; ^16^ Department of Urology Copenhagen University Hospital—Zealand University Hospital Roskilde Roskilde Denmark; ^17^ Department of Clinical Medicine University of Copenhagen Copenhagen Denmark; ^18^ Department of Urology University Hospital of Rion Patras Greece; ^19^ Department of Urology University Hospital Southampton Southampton UK; ^20^ Department of Urology UROSUD, La Croix Du Sud Hospital Quint‐Fonsegrives France; ^21^ Nuffield Department of Surgical Sciences University of Oxford Oxford UK; ^22^ Department of Urology Assistance Publique—Hôpitaux de Paris, Tenon Academic Hospital, Sorbonne University Paris France; ^23^ Center for Surgery and Public Health, Department of Surgery Brigham and Women's Hospital Boston Massachusetts USA; ^24^ Division of Trauma Emergency Surgery and Surgical Critical Care, Department of Surgery Massachusetts General Hospital Boston Massachusetts USA

**Keywords:** policy, pregnancy, urology training

## Abstract

**Background:**

There is no information regarding challenges faced by European urology residents in starting a family. The aim of this study was to examine pregnancy and parenting experiences and policies during urology residency, identify regulatory gaps and provide recommendations for standardized European guidelines.

**Methods:**

A cross‐sectional English‐language electronic survey targeted urology residents and young urologists across Europe. The 44‐item, self‐administered survey was pilot‐tested and iteratively revised with European Society of Residents in Urology (ESRU) board members. Data were collected between August and October 2024 from ESRU, the European Association of Urology (EAU) and the European School of Urology (ESU) via mailing lists and social media platforms.

**Results:**

From 387 respondents, 237 (61%) were females and 255 (66%) were residents. Written policies on pregnancy and parenthood management were reported by 112 (29%) respondents. Formal discussions on pregnancy and parenting were reported to be absent from 319 (82%) respondents, though 228 (59%) agreed about their importance. Among 250 non‐childbearing participants, parenthood was postponed due to fears of missing training opportunities in 130 (92%) female versus 76 (86%) male respondents and due to fears of missing career opportunities in 122 (86%) female versus 62 (70%) male respondents. Concerns about falling behind peers in training were present in 119 (84%) female versus 58 (66%) male respondents (*p* < 0.001). Across all participants, 283 (87%) and 277 (85%) supported adjusting residency working and training schedules, respectively, upon return to work to ensure the completion of residency requirements. Both male and female respondents strongly endorsed standardized European guidelines on pregnancy and parenting in urology residency.

**Conclusions:**

This survey highlights the significant barriers to family planning in European urology residency. Fear of career setbacks and training disruptions drives parenthood delays. Standardized policies are needed to support residents while maintaining training requirements and career progression during pregnancy and parenthood.

## 1 INTRODUCTION

As the representation of females in medical training continues to rise across Europe,^1^, ^2^, ^3^ a strong imbalance persists within surgical specialization,^3^ with women representing only 27% of the surgical workforce in the United Kingdom.^4^ There have been numerous studies documenting challenges US surgeons face in starting a family, with regulatory bodies mandating only 6 weeks of parental leave.^5^ However, less is known about parenthood among surgeons in Europe, where the European Union (EU) Work–Life Balance Directive provides a minimum of 4 months of leave per parent, of which at least 2 months are paid and non‐transferable (compensated at a level to be set by member states).^6^ Within surgical specialties, urology has historically been a predominantly male field.^7^, ^8^ This is driven by perception of gender discrimination, academic disparities, training differences in comparison with the male counterpart, and an incompatibility between rigorous surgical training and family life aspirations.^8^

Challenges related to pregnancy during training have been identified as one of the major challenges faced by female physicians and a reason for not choosing a surgical specialty. Concerns about family planning during surgical residency represent a fundamental topic for medical students when choosing their specialty.^9^ Reports indicate that nearly 40% of residents consider leaving residency while pregnant, and a substantial 30% would not recommend others to pursue a surgical career.^10^ A UK survey revealed that 28% of urology trainees suffered miscarriages, a proportion exceeding that in the general population, and 35% encountered severe obstetric or postnatal complication.^11^ The decision to postpone childbearing, largely influenced by fear of missing essential training and academic opportunities, intensifies risks for pregnancy‐related health conditions.^5^, ^10^ Furthermore, urological practice often involves exposure to potential risks including ionizing radiation, prolonged surgeries, and irregular hours involving weekend and night shifts.

Despite the existence of the aforementioned EU Work–Life Balance Directive, the translation of these labour protection measures into consistent hospital‐ and department‐level policies, particularly within surgical specialties, remains heterogeneous. Europe lacks standardized guidelines addressing residency training during pregnancy and parenthood such as protection from hazardous exposures, regulated work hours or procedures for equitable reintegration post‐parental leave.^12^ Such disparities pose significant challenges to harmonizing work–life balance within surgical specialties. In light of these observations, this survey was designed to systematically capture the perspectives of European residents and urologists and to examine existing institutional policies governing pregnancy and parenthood during urology residency across Europe.

## 2 METHODS

### 2.1 Survey design and development

A descriptive, cross‐sectional survey was designed using a cognitive testing approach to collect comprehensive data on pregnancy and parenthood policies in European urology residency programmes and across European countries.^13^ A 44‐item self‐administered online questionnaire was developed by the European Society of Residents in Urology (ESRU) board and administered exclusively in English, without translated versions (see Data S1 for the residents' survey and Data S2 for the consultants' survey). The survey content was defined by a focused literature review on the following key terms: *residency*, *parenthood*, *pregnancy*, *surgery* and *regulations*. Moreover, current EU policies were evaluated to ensure that the questions align with international regulations.^14^ The survey was structured to contain the following domains: demographic information; family goals; views on pregnancy and parenthood from childbearing and non‐childbearing participants; regulations on pregnancy, maternity and paternity leave, and return to work; and views on general European recommendations about the management of pregnancy and parenthood during urology residency. For clarity, ‘non‐childbearing participants’ were defined as respondents who had not yet had children at the time of survey completion, regardless of future intentions or desire to have children, and this term does not imply voluntary or permanent childlessness.

Content expertise was provided by experts specializing in surgical training and education, pregnancy and parenthood policy, and survey design. Multinational expert focus groups including senior urology residents, urologists, professionals from various surgical branches and experts in survey design were assembled. The international composition of the group was essential for crafting questions that would be understandable and interpretable by a diverse audience with different cultural backgrounds, native languages and residency structures. Question development took place from October 2023 to March 2024. The survey was iteratively tested and revised for clarity and relevance from April 2024 to July 2024. This process ensured content validity by ruling out inconsistencies, errors or ambiguities during the final evaluation rounds.

### 2.2 Data collection

The survey was disseminated electronically between August and October 2024 through ESRU, the European Association of Urology (EAU) and the European School of Urology (ESU). Mailing lists, social media platforms and institutional channels of ESRU, EAU and ESU were utilized to obtain broad reach. To incentivize participation, reminders were sent every 10 days via social media or mailing lists. Owing to the descriptive and exploratory design of the survey, no a priori power or sample size calculation was undertaken; instead, the sample size was determined by feasibility and convenience, with the survey disseminated broadly to maximize participation.

The unit of analysis included both female and male urology residents working in an EU country, Switzerland and the United Kingdom. The unit of observation comprised urology residents and young urologists. Participation was voluntary, with respondents providing implied consent by completing the survey.

Eligibility criteria included active or past urology training in Europe or current urology practice within Europe. Respondents who neither completed their urology residency in Europe nor worked in a European facility at the time of survey completion were excluded. The SurveyMonkey^®^ (Survey Monkey Inc., San Mateo, CA, USA) platform was selected due to its user‐friendly design, accessibility, ability to detect duplicate survey responses and ease of data export for subsequent analysis with commonly used statistical software.^15^

### 2.3 Objectives

The aims of the study were to capture current pregnancy‐related policies and personal experiences regarding pregnancy and parenthood policies among urology residents and young urologists across Europe, identify gaps in existing regulations, assess their impact on training and provide recommendations for standardized European guidelines.

### 2.4 Data analysis

Statistical analysis was performed by stratifying according to gender and training status. Qualitative responses were categorized thematically. Categorical variables were reported as frequencies and proportions and analysed using either the chi‐squared or Fisher's exact test. Medians and interquartile ranges (IQRs) were recorded for continuously coded variables. Data from continuous variables were assessed for normality using the Shapiro–Wilk test. Parametric tests (two‐sample independent *t* test) were used for normally distributed continuous variables, while non‐parametric tests (Wilcoxon rank‐sum test) were applied to non‐normally distributed data and ordinal variables. All statistical analyses were performed using the R software (Version 4.4.3, R Foundation for Statistical Computing, Vienna, Austria, 2020). All reported *p* values are two‐sided, with a threshold of *p* ≤ 0.05 considered statistically significant.

## 3 RESULTS

**FIGURE 1:**
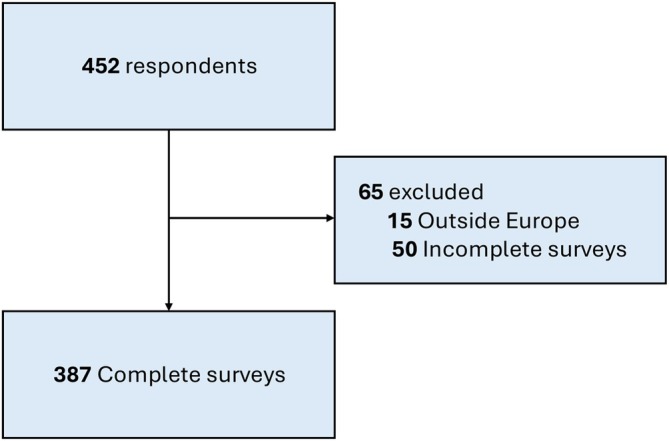
CONSORT diagram depicting the selection process of the participants responding to the survey on pregnancy and parenting from each European country.

**FIGURE 2:**
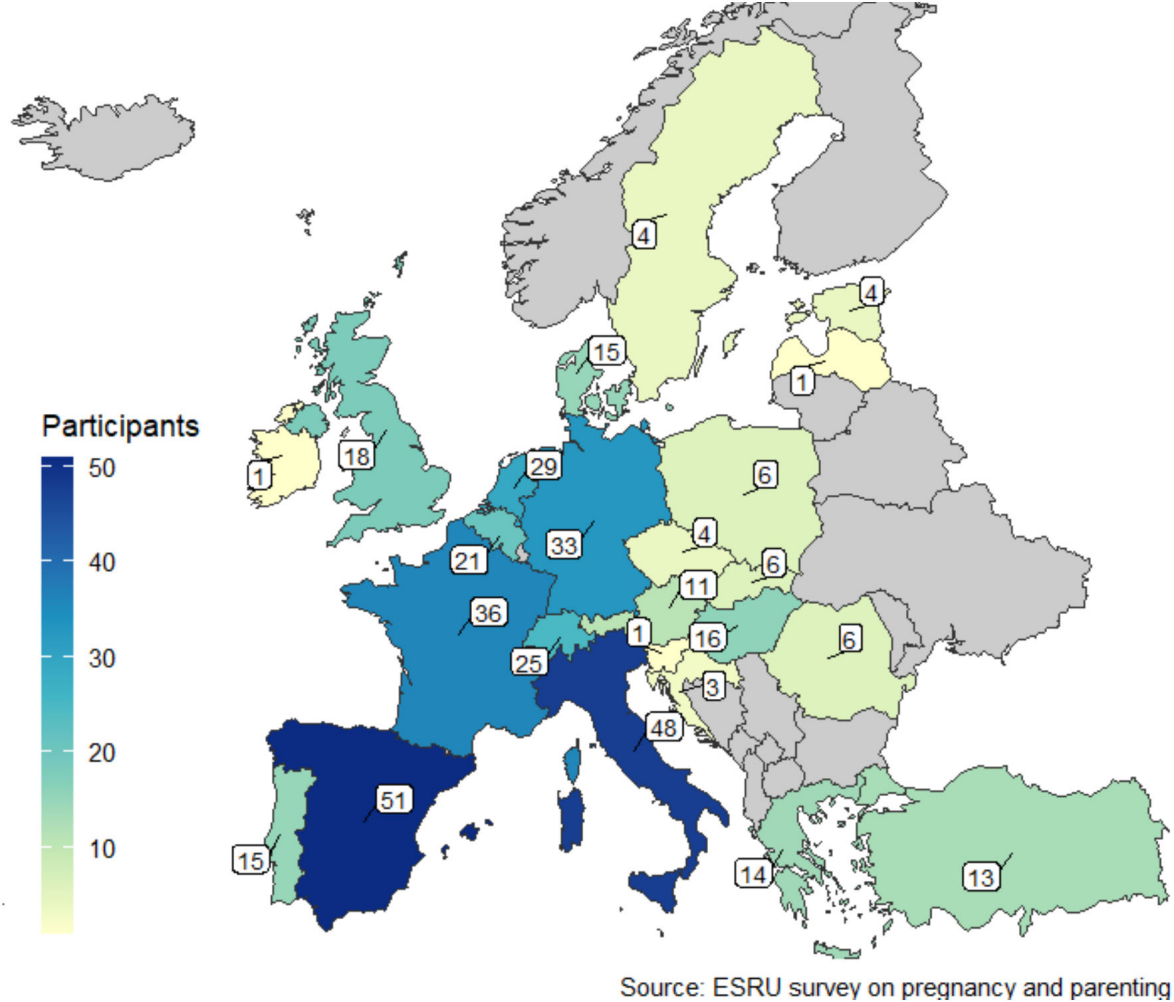
European map displaying the number of participants responding to the survey on pregnancy and parenting from each European country.

From a total of 1315 emails sent, 452 responses were collected (response rate 34.4%). After removing incomplete surveys and responses from outside Europe, 387 complete surveys from participants working in European countries were included in the final analysis (Figure 1). Participants represented all EU countries except Ireland, Finland, Norway, Estonia and Macedonia. The highest number of responses came from Italy, Spain and France (Figure 2). Luxembourg and Iceland were excluded due to the absence of urology residency programmes.

### 3.1 Sociodemographic characteristics

Among the 387 respondents, 237 (61%) were female, 255 (66%) were residents, and 207 (53%) were aged 30–34 years (Table 1). Most responses (155, 40%) were collected from Western Europe (i.e., Austria, Belgium, France, Germany, the Netherlands and Switzerland), followed by Southern Europe (i.e., Albania, Croatia, Greece, Italy, Macedonia, Portugal, Serbia, Slovenia, Spain and Turkey [149, 38%]) (see Figure 2). While 204 (80%) respondents were married or in a relationship (Table 2), male respondents were less commonly in a relationship with a physician or a resident compared to their female counterparts (27% vs. 59%, *p* < 0.001).

**TABLE 1:** Sociodemographic data of respondents. Statistically significant differences are indicated by bold *p* values.

Variables	Overall (*N* = 387, 100%)	Male urologists (*N* = 150, 39%)	Female urologists (*N* = 237, 61%)	*p* value
Age (years), *n* (%)				
≤29	82 (21.2)	28 (18.7)	54 (22.8)	0.9
30–34	207 (53.5)	88 (58.7)	119 (50.2)	
35–39	79 (20.4)	27 (18)	52 (21.9)	
≥40	19 (4.9)	7 (4.7)	12 (5)	
Training status, *n* (%)				
Resident	255 (66)	94 (63)	161 (68)	0.2
Young urologists	132 (34)	56 (37)	76 (32)	
Years of training (residents), *n* (%)				
1–2	38 (14.9)	7 (7.4)	31 (19.3)	0.4
3–5	169 (66.3)	67 (71.3)	102 (63.4)	
6–10	48 (18.8)	20 (21.3)	28 (17.4)	
Years since end of residency (young urologists), *n* (%)				
1–2	56 (42.4)	25 (44.6)	31 (40.8)	0.3
3–4	41 (31.1)	18 (32.1)	23 (30.3)	
5–6	35 (26.5)	13 (23.2)	22 (28.9)	
European working area^a^, *n* (%)				
Southern Europe	149 (38.5)	80 (33.8)	69 (46)	0.08
Western Europe	155 (40.1)	101 (42.6)	54 (36)	
Northern Europe	43 (11.1)	31 (13.1)	12 (8)	
Eastern Europe	40 (10.3)	25 (10.5)	15 (10)	
Working hours per week, *n* (%)				
≤30	17 (4.4)	5 (3.3)	12 (5.1)	0.1
31–50	130 (33.6)	41 (27.3)	89 (37.6)	
51–70	183 (47.3)	77 (51.3)	106 (44.7)	
≥71	57 (14.7)	27 (18)	30 (12.7)	
Practice setting^b^, *n* (%)				
University hospital	270 (69.8)	114 (76)	156 (65.8)	**0.03**
Non‐university hospital	110 (28.4)	34 (22.7)	76 (32.1)	
Non‐academic hospital	7 (1.8)	2 (1.3)	5 (2.1)	
Future practice setting^c^, *n* (%)				
Hospital, academic track	193 (49.9)	78 (52)	115 (48.5)	0.5
Hospital, non‐academic track	170 (43.9)	62 (41.3)	108 (45.6)	
Private practice	23 (5.9)	9 (6)	14 (5.9)	
Other	1 (0.3)	1 (0.7)	0	
Subspecialty (aimed at or practised), *n* (%)				
General urology	55 (14.2)	16 (10.7)	39 (16.5)	0.2
Neurourology	6 (1.6)	2 (1.3)	4 (1.7)	
Endourology	58 (15)	27 (18)	31 (13.1)	
Urologic oncology	169 (43.7)	78 (52)	91 (38.4)	
Urologic trauma and reconstructive urology	21 (5.4)	8 (5.3)	13 (5.5)	
Paediatric urology	12 (3.1)	4 (2.7)	8 (3.4)	
Male sexual and reproductive health	20 (5.2)	7 (4.7)	13 (5.5)	
Renal transplantation	4 (1)	2 (1.3)	2 (0.8)	
Urogynecology	12 (3.1)	0	12 (5.1)	
Uncertain	30 (7.8)	6 (4)	24 (10.1)	

^a^ According to the *United Nations geoscheme*: Southern Europe: Albania, Croatia, Greece, Italy, Macedonia, Portugal, Serbia, Slovenia, Spain and Turkey; Western Europe: Austria, Belgium, France, Germany, the Netherlands and Switzerland; Northern Europe: Denmark, Estonia, Ireland, Latvia, Sweden and the United Kingdom; and Eastern Europe: Czech Republic, Georgia, Hungary, Poland, Romania and Slovakia.

^b^ Practice setting: university hospital = associated with a medical school, with urology residency; non‐university hospital = not associated with a medical school, with urology residency; and non‐academic hospital = community hospital, without medical school and without urology residency.

^c^ Future perspective: academic track = combining clinical/surgical practice with teaching/research; non‐academic track = focusing on clinical/surgical practice; and other = industry, administration and research.

**TABLE 2:** Relationship status of residents participating in the survey. Statistically significant differences are indicated by bold *p* values.

Item	Overall (*N* = 255, 100%)	Male urologists (*N* = 94, 37%)	Female urologists (*N* = 161, 63%)	*p* value
Relationship status, *n* (%)				
Single	51 (20)	15 (16)	36 (22.3)	0.9
Married	92 (36.1)	35 (37.2)	57 (35.4)	
In a relationship	112 (43.9)	44 (46.8)	68 (42.2)	
Relationship setting^a^, *n* (%)				
Living in the same household	171 (83.8)	66 (83.5)	105 (84)	0.2
Short‐distance relationship^b^	18 (8.8)	4 (5.1)	14 (11.2)	
Long‐distance relationship^c^	15 (7.4)	9 (11.4)	6 (4.8)	
Professional position of the partner, *n* (%)				
Employed but not as a physician or resident	103 (50.5)	29 (36.7)	74 (59.2)	**<0.001**
Specialized physician or resident in a medical field	60 (29.4)	37 (46.8)	23 (18.4)	
Specialized physician or resident in a surgical field	35 (17.2)	9 (11.4)	26 (20.8)	
Not employed	6 (2.9)	4 (5.1)	2 (1.6)	
Working hours per week (partner), *n* (%)				
≤30	21 (10.3)	13 (16.5)	8 (6.4)	0.08
31–50	121 (59.3)	43 (54.4)	78 (62.4)	
51–70	56 (27.5)	22 (27.8)	34 (27.2)	
≥71	6 (2.9)	1 (1.3)	5 (4)	

^a^ Includes married/in a relationship respondents.

^b^ Relationship in the same city, but in a different household.

^c^ Relationship across different cities or countries.

### 3.2 Views of childbearing parents

**FIGURE 3:**
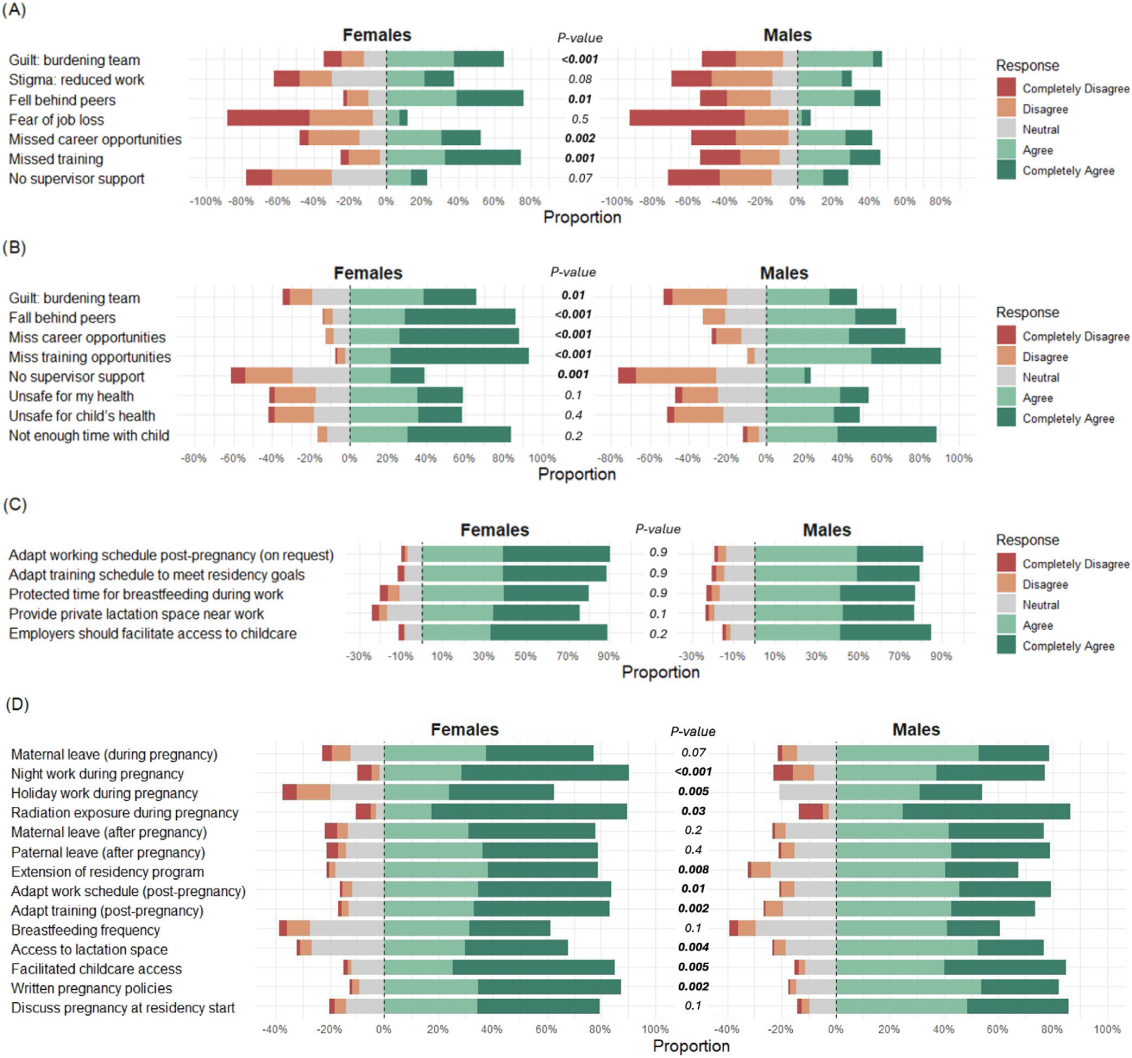
Diverging stacked bar plot illustrating the views of residents and young urologists in urology on different topics related to pregnancy and parenting. *p* values indicate differences between agree/completely agree grouped together and disagree/completely disagree grouped together according to sex. (A) Perceived stigma and support during pregnancy (childbearing participants), (B) concerns about having a child during residency (non‐childbearing participants), (C) agreement or disagreement with institutional accommodations after pregnancy and (D) agreement on the necessity of recommendations for managing pregnancy during urology residency.

Among the 157 participants who had children during residency, female respondents were significantly more likely to take unpaid parental leave during pregnancy compared to the male counterpart (34% vs. 10%; *p* = 0.02) (Table 3). Similarly, 48 (50%) of the childbearing female respondents reduced their work schedules, a measure taken by only 3 (5%) of the childbearing male participants (*p* < 0.001). On average, female childbearing participants began reducing their workload around the 24th week of pregnancy (IQR: 4–36 weeks); 59 (62%) childbearing female respondents reported feelings of guilt about burdening colleagues by reducing their workload or call hours, compared to 18 (29%) childbearing male respondents (*p* < 0.001) (Figure 3A). Similarly, 46 (48%) childbearing females expressed concerns about missing career opportunities, in contrast to 17 (27%) childbearing males (*p* = 0.002); 69 (73%) childbearing female respondents also felt that they missed training opportunities, in comparison with 19 (31%) childbearing male respondents (*p* = 0.01).

**TABLE 3:** Children, parenthood timing, and working hours for participants who were pregnant or parents. Statistically significant differences are indicated by bold *p* values.

Item	Overall (*N* = 157, 100%)	Male urologists (*N* = 62, 40%)	Female urologists (*N* = 95, 60%)	*p* value
Children, *n* (%)				
1	100 (63.7)	42 (67.7)	58 (61.1)	0.5
2	43 (27.4)	15 (24.2)	28 (29.5)	
3+	14 (8.9)	5 (8.1)	9 (9.5)	
Parenthood timing, *n* (%)				
Before residency	6 (3.8)	3 (4.8)	3 (3.2)	0.6
During residency	103 (65.6)	42 (67.7)	61 (64.2)	
After residency	9 (5.7)	2 (3.2)	7 (7.4)	
Before and during residency	36 (22.9)	13 (21)	23 (24.2)	
During and after residency	2 (1.3)	1 (1.6)	1 (1.1)	
Before, during and after residency	1 (0.6)	1 (1.6)	0	
Need to take a break from residency, *n* (%)				
No	58 (36.9)	32 (51.6)	26 (27.4)	**0.02**
Unpaid parental leave	38 (24.2)	6 (9.7)	32 (33.7)	
Research, PhD and master's	5 (3.2)	3 (4.8)	2 (2.1)	
None of the above	56 (35.7)	21 (33.9)	35 (36.8)	
Average hours per week worked during pregnancy^a^, *n* (%)				
≤30	39 (29.3)	7 (16.7)	32 (35.2)	**0.005**
31–50	78 (58.6)	25 (59.5)	53 (58.2)	
51–70	16 (12)	10 (23.8)	6 (6.6)	
Average week to reduce working schedule during pregnancy, median (IQR)	24 (4–36)	‐	24 (4–36)	0.6
Need to reduce working schedule during pregnancy, *n* (%)	51 (32.5)	3 (4.8)	48 (50.5)	**<0.001**

^a^ For males, it identifies the average number of hours worked by them, not by their partners.

### 3.3 Views of non‐childbearing parents

The perspective of 250 non‐childbearing respondents during residency is illustrated in Figure 3B. Parenthood was postponed due to fears of missing training opportunities in 130 (92%) female versus 76 (86%) male respondents and due to fears of missing career opportunities in 122 (86%) female versus 62 (70%) male respondents (both *p* < 0.001). Concerns about falling behind peers in training were present in 119 (84%) female versus 58 (66%) male respondents (*p* < 0.001). Concerns over burdening colleagues with additional work or call hours and over not receiving enough support from supervisors were prevalent among female respondents (65% vs. 45%, *p* = 0.01, and 38% vs. 23%, *p* = 0.001). Concerns of having insufficient time for childcare were a major reason for postponing parenthood in both sexes (82% for female and 84% for male respondents).

### 3.4 Discussion about the management of pregnancy during residency

Formal discussions on pregnancy and parenting at the beginning of residency did not take place for 319 (82%) respondents, though 228 (59%) agreed or strongly agreed that it would have been important (Figure S1). Moreover, only 112 (30%) respondents reported about the presence of a written internal policy regarding the management of pregnancy during residency.

### 3.5 National regulations on the management of pregnancy and parenthood

While 159 (41%) respondents indicated that there is a mandatory requirement to avoid ionizing radiation exposure during pregnancy, 36 (9.3%) reported the absence of any formal guideline (Table 4). Participation in surgical procedures also lacks consistency; 72 (18%) respondents stated that participation in any surgical procedure is allowed, whereas 74 (19%) noted that there are no established regulations. Residency extensions due to childbearing parental leave are reported by 226 (58%) respondents, yet only 113 (29%) indicated that such extensions are mandated for non‐childbearing parental leave. Upon returning to work, official adaptations to work schedules are reported to be regulated by 93 (24%) respondents, whereas 166 (43%) mentioned no applicable regulations exist. Training schedule adaptations to meet residency objectives are rarely addressed, with only 20 (5%) reporting the presence of official rules and 221 (57%) noting a complete absence of regulations. Lactation breaks were regulated for 117 (30%) respondents, and only 69 (18%) reported the presence of private lactation space requirements. Childcare support regulations are scarce, with only 37 (9.6%) residents reporting that their programme requires employers to facilitate childcare access. To illustrate the heterogeneity in legal frameworks across Europe, we compiled country‐specific data on maternity, paternity and parental leave, as well as radiation protection and workplace accommodations for pregnant healthcare workers (Table S1).

**TABLE 4:** National regulations regarding different aspects of pregnancy and parenting during urology residency in Europe.

Item	Overall (*N* = 387, 100%)
Regulation concerning exposure to ionizing radiations, *n* (%)	
Exposure is permitted	33 (8.5)
Exposure should be avoided	77 (19.9)
Exposure must be avoided	159 (41.1)
There is no official regulation	36 (9.3)
I don't know	56 (14.5)
Missing	26 (6.7)
Regulation concerning participation to surgical procedures (as operator or assistant), *n* (%)	
It is prohibited to participate in all surgical procedures	25 (6.5)
It is prohibited to participate in some surgical procedures	28 (7.2)
It is recommended to avoid participation in all surgical procedures	18 (4.7)
It is recommended to avoid participation in some surgical procedures	72 (18.6)
It is allowed to participate to any surgical procedure	72 (18.6)
There is no official regulation	74 (19.1)
I don't know	72 (18.6)
Missing	26 (6.7)
Requirement to extend the duration of urology residency because of childbearing parental leave, *n* (%)	
No	17 (4.4)
Yes	226 (58.4)
It depends	69 (17.8)
I don't know	37 (9.6)
Missing	38 (9.8)
Requirement to extend the duration of urology residency because of non‐childbearing parental leave, *n* (%)	
No	122 (31.5)
Yes	113 (29.2)
It depends	45 (11.6)
I don't know	69 (17.8)
Missing	38 (9.8)
Presence of a regulation concerning the adaptation of the working schedule (e.g., reduction to part‐time) upon return to work, *n* (%)	
Yes, there is an official regulation	93 (24)
No, there is no official regulation	166 (42.9)
I don't know	79 (20.4)
Missing	49 (12.7)
Presence of a regulation concerning the adaptation of the training schedule to achieve planned urology residency objectives upon return to work, *n* (%)	
Yes, there is an official regulation	20 (5.2)
No, there is no official regulation	221 (57.1)
I don't know	97 (25.1)
Missing	49 (12.7)
Legal regulation on the frequency of breastfeeding breaks during working hours, *n* (%)	
Yes, there is an official regulation	117 (30.2)
No, there is no official regulation	127 (32.8)
I don't know	94 (24.3)
Missing	49 (12.7)
Requirement of the employers to provide a private lactation space in proximity to work environment, *n* (%)	
Yes	69 (17.8)
No	151 (39)
I don't know	118 (30.5)
Missing	49 (12.7)
Requirement of the employers to facilitate the working environment towards access to childcare, *n* (%)	
Yes	37 (9.6)
No	180 (46.5)
I don't know	121 (31.3)
Missing	49 (12.7)

### 3.6 Institutional accommodations after pregnancy

The majority of 387 respondents expressed agreement with supportive workplace policies after pregnancy (Figure 3C). The adaptation of the working schedule upon return to work was endorsed by 181 (76%) female versus 102 (68%) male participants, while adaptation of the training schedule was supported by 177 (79%) female and 100 (67%) male participants. Agreement with providing a private lactation space was reported by 151 (64%) female and 92 (61%) male respondents. Similarly, protected time for breastfeeding during working hours was supported by 159 (64%) females and 94 (63%) males. Support for facilitated access to childcare was high among both groups, with 178 (75%) females and 105 (70%) males indicating agreement.

### 3.7 European recommendations for the management of pregnancy and parenthood

Figure 3D illustrates the view across both sexes regarding the need for harmonized, Europe‐wide policies on topics related to pregnancy and parenthood during residency. Overall, both female and male respondents showed high levels of agreement with the necessity of developing European recommendations related to managing pregnancy during urology residency, though some sex‐based differences in the degree of support were evident. In terms of agreement rates, 90.3% of females expressed the strongest consensus for establishing clear regulations on night work restrictions, 89.5% on radiation protection during pregnancy and 87.2% on the need for internal pregnancy‐related policies. Similarly high levels of agreement were reported for facilitated childcare access (85%), adaptation of working schedules (83%) and adaptation of training schedules (83.6%) upon return to work. Additionally, over 70% of female respondents agreed with recommendations to discuss pregnancy at the beginning of residency (79.7%), maternity leave during pregnancy (78%), maternity leave after pregnancy (77.3%) and paternity leave after pregnancy (79%).

Among male residents, the highest levels of agreement were for radiation protection (86.4%), internal policies (82.4%) and facilitated childcare access (84.8%). Other recommendations with substantial male support included adaptation of working schedules (79.2%), adaptation of training schedules (73.3%), discussion of pregnancy at the beginning of residency (85.7%) and paternity leave after pregnancy (79%). Maternity leave during pregnancy was also well supported by males, with 78.8% in agreement.

### 3.8 Results stratified according to training status

Data stratified by training status are reported in Table S2. Consultants were significantly older than residents, with most aged 30–39 years (85.6% vs. 67.8%, *p* < 0.001). Employment in university hospitals was common in both groups but more frequent among consultants (79.5% vs. 64.7%, *p* = 0.007), who were also more likely to follow an academic track (68.2% vs. 40.4%, *p* < 0.001). Parenthood was more prevalent among consultants, with 70 (53%) reporting one or more children compared with 87 (34%) residents (*p* < 0.001). Among parents, residents more often reported parenthood before residency (31.8%), whereas consultants more frequently reported parenthood after completion of training (27.3%).

As shown in Table S3, only a minority of respondents reported the presence of a written institutional policy on pregnancy management during residency (27.8% of residents vs. 31.1% of consultants, *p* = 0.7). In both groups, pregnancy or parental leave management was rarely discussed with supervisors (81.6% vs. 84.1%, *p* = 0.8), despite more than half of respondents considering such discussions to be very or quite important.

Among participants with children (Figure S2A), perceptions of stigma, guilt and career impact were common and comparable between groups. Feelings of guilt related to burdening colleagues were reported by 48.6% of consultants and 49.4% of residents; concerns about missed training opportunities were reported by 52.9% and 58.6%, respectively; and 57.1% versus 56.3% reported having fallen behind their peers. Among non‐childbearing respondents (Figure S2B), anticipated training and career impact was even more pronounced, with nearly all respondents expecting missed training opportunities (89.3% of residents and 90.3% of consultants) and a large majority anticipating missed career opportunities (81% vs. 77.4%). Regarding return‐to‐work conditions after pregnancy (Figure S2C), agreement on the need for adapted work and training schedules, availability of lactation facilities, protected breastfeeding time and facilitated access to childcare ranged from 75% to 88%, without significant differences by training status. Finally, most residents and consultants supported the need for European‐level regulations on pregnancy and parenthood management during urology residency (Figure S2D), again without significant differences between groups.

## 4 DISCUSSION

This study highlights significant gaps in the management of pregnancy and parenthood within European urology residency programmes. Female residents reported significantly higher levels of concern regarding potential setbacks in training and career opportunities due to pregnancy, which were less pronounced among their male counterparts. This aligns with previous research highlighting the gender‐specific challenges in medical training, where female trainees often experience greater difficulty balancing professional and personal responsibilities.^16^, ^17^, ^18^, ^19^ Moreover, females were more likely to perceive an increased burden on colleagues and a lack of support from supervisors during pregnancy and parenthood transitions. These gender‐based differences underscore the need for tailored policies.^20^ Notably, perceptions regarding stigma, concerns about training and career impact, and the need for institutional accommodations and standardized regulations did not differ between residents and consultants, indicating that concerns related to pregnancy, parenthood and institutional support persist and are equally relevant across training stages. Furthermore, the management of pregnancy and parenthood is rarely addressed during residency, despite being strongly desired by trainees. To address this gap, a simple checklist with predefined questions may serve as a practical tool to facilitate structured discussions and institutional planning (see Table S4 for an example).

Our study uncovers that in Europe, challenges are similar to US surgeons.^5^, ^10^ One of the main similarities between our study and previous US findings is that extended leaves without supportive reintegration systems can lead to significant training interruptions, which are feared by residents.^5^, ^10^, ^21^ The career trajectory of childbearing trainees in the medical field is influenced not merely by the time taken off for childbirth but more significantly by the subsequent reintegration process into the workforce. As highlighted in our study, urology trainees often delay pregnancy and parenthood because of fear of mistreatment and stigma, fear of not being supported by their colleagues, and fear of missing career and training opportunities. While policy frameworks support various degrees of parental leave across Europe and the world,^5^ the reality of reintegration can vary, potentially stalling the careers of those who temporarily step away from their professional paths. This underscores the importance of supportive measures that ensure equal access to educational and professional opportunities for all trainees, irrespective of their personal circumstances.

While formal regulations addressing occupational hazards during pregnancy often exist at the national or supranational level, our findings suggest that inconsistent implementation and limited departmental awareness remain major challenges in daily clinical practice. The variability observed in radiation protection practices indicates that the primary limitation lies not in the absence of regulation, but in fragmented dissemination, local enforcement and prevailing cultural norms within surgical departments. This disconnection between formal policy and clinical reality may contribute to persistent safety concerns and reinforce trainees' uncertainty.

Without structured policies, trainees—especially females—face career progression delays and increased stress, impacting mental health and family planning decisions.^17^ Standardized European guidelines could address these issues by ensuring equitable parental leave, safe working conditions during pregnancy, and access to necessary support facilities like lactation spaces and childcare. To achieve gender equality in surgical training, policies must be reformed to provide equitable parental leave options for all residents, ensuring that both males and females have access to sufficient leave without disproportionately impacting career progression. If childcare is no longer considered the sole domain of females and more fathers take parental leave, the outcomes for gender equality could include increased female labour‐market participation, reduced gender pay gaps and increased male participation in household work.^22^ Implementing paid, standardized parental leave for all genders, along with flexible training structures and institutional support, would help mitigate the career delays faced primarily by females and foster a more inclusive and fair surgical training environment.

By establishing comprehensive frameworks, residency programmes could help trainees manage personal commitments without compromising their careers, thereby maintaining rigorous training standards and fostering gender equity.^23^ Our survey suggests several targeted recommendations for policymakers: (1) defining standardized leave policies (clear, equitable parameters for childbearing and non‐childbearing parental leave), (2) creating clear internal guidelines (outlining institutional expectations and support mechanisms for pregnant and parenting trainees), (3) fostering safe work conditions (excluding hazardous duties during pregnancy and providing flexible work schedules for smooth reintegration post‐leave) and (4) improving support facilities (ensuring the availability of lactation spaces and childcare services to alleviate logistical challenges). Extending these recommendations to fellowship training might be meaningful, considering that previous studies have shown that family planning during fellowship is not supported and might lead to personal and professional dissonance.^24^

Beyond addressing the evident gaps in formal policy frameworks, the imperative of mentorship in facilitating work–family integration for female urologists warrants significant emphasis. As females comprise a minority within the field of urology,^7^ female residents and young urologists frequently encounter challenges in identifying role models and mentors who can impart experience‐based guidance on harmonizing professional and familial obligations.^25^ This paucity of mentorship resources can render female urologists vulnerable to navigating these complexities without adequate support. Recent literature underscores mentorship's critical role in promoting both career development and work–life balance. For instance, Moore et al. indicate that mentorship programmes can structure essential support systems, enabling trainees to articulate concerns and obtain bespoke advice for addressing the intricate interplay of personal and professional challenges.^25^ Mentorship could also improve career satisfaction among female surgeons, thereby fostering an inclusive and supportive training milieu.^26^ Integrating structured parental mentorship initiatives within the ESRU and EAU frameworks could represent a meaningful pilot strategy.

While this study is the first to provide insights into the management of pregnancy and parenthood within European urology residency programmes, some limitations must be acknowledged. First, the reliance on self‐reported data in the survey design may introduce recall bias. Second, the disproportionate female participation likely reflects greater engagement with pregnancy‐ and parenthood‐related topics among female urologists rather than the actual demographic distribution within the European urologic workforce. Consequently, a potential non‐response bias cannot be excluded, as male residents and urologists may have been less inclined to complete a survey focusing on pregnancy and parenthood. This overrepresentation should therefore be considered when interpreting findings and limits the generalizability of gender‐stratified analyses. Underrepresentation of certain countries may restrict the generalizability of the findings. Moreover, the sample size is limited and does not permit state‐specific analysis. Furthermore, the cross‐sectional nature of the survey provides only a snapshot in time and does not track changes or trends over time. This limits our ability to infer causality or the long‐term effects of the identified policy gaps on trainees' career progression and personal life. In addition, this study focused exclusively on European regulatory frameworks, and comparisons with non‐European guidelines were beyond its scope.

To address these limitations, longitudinal studies tracking trainees over time would be fundamental in evaluating the impact of implemented guidelines on career development, work–life balance and well‐being, offering robust evidence of policy effectiveness. Additionally, qualitative research through interviews or focus groups would offer deeper insights into the personal experiences of trainees managing pregnancy and parenthood during residency. Future research could also incorporate comparisons with non‐European regulatory frameworks to contextualize European policies within a broader international perspective. Lastly, future surveys should also seek to capture the perspectives of gender‐diverse and non‐binary trainees, as inclusivity in policy development is essential to ensure equitable support for all individuals in surgical training. These research efforts would play a crucial role in helping to develop evidence‐based guidelines and enhancing support systems for trainees in surgical and other medical fields.

## 5 CONCLUSIONS

This study highlights significant gaps in policies supporting pregnancy and parenthood within European urology residency programmes, with female trainees facing notable disparities. Despite existing parental leave frameworks, inconsistencies in implementation and reintegration support exist, exacerbating gender‐specific challenges. Standardized European guidelines are urgently needed to provide equitable parental leave, safe working conditions during pregnancy and essential support facilities. Tailoring these policies to cultural contexts and emphasizing mentorship can create an inclusive environment that supports trainees' professional and personal needs.

## AUTHOR CONTRIBUTIONS

*Study concept and design*: Luca Afferi, Benjamin Pradere, Laura S. Mertens, Joel S. Weissman, Erika Rangel. *Data acquisition*: Luca Afferi, Gaelle Margue, Carlos Toribio‐Vázquez, Belle M. Melsert, Loic Baekelandt, Anna Goujon, Mehmet Ozalevli, Enrico Checcucci, Anna Gugerli Lazos, Carolin Siech, Juan Luis Vásquez, Laura S. Mertens, Evangelos Liatsikos, Bhaskar K. Somani, Benjamin Pradere. *Statistical analysis*: Luca Afferi. *Drafting of the manuscript*: Luca Afferi, Gaelle Margue, Carlos Toribio‐Vázquez, Belle M. Melsert. *Critical revision of manuscript*: All authors.

## ACKNOWLEDGEMENTS

The authors would like to thank the Research Fund of the Hospital of Aarau for covering the publication costs, as well as all participants of the survey.

## CONFLICT OF INTEREST STATEMENT

The authors declare no conflicts of interest.

## Supporting information


**Data S1.** Supporting Information

**Data S2.** Supporting Information

**Figure S1.** Pie chart illustrating the importance for residents to discuss with their boss or supervisor how pregnancy/parenting are managed in their department.
**Table S1.** European Country‐Level legal protections on pregnancy and parenthood. Hereby we present a list of maternity leave, paternity leave, parental leave, radiation protection for pregnant workers, workplace accommodations (lactation rooms, breastfeeding breaks, childcare), and post‐leave schedule/residency rules for most European countries. Medical training/residency context is prioritized where applicable. Data reflect current frameworks circa 2024–2025; see sources in the last column for each country.
**Table S2.** Sociodemographic data of respondents after stratification by training status.
**Table S3.** Internal regulations regarding management of pregnancy during pregnancy after stratification by training status.
**Figure S2**. Diverging stacked bar plot illustrating the views of residents and consultants in urology on different topics related to pregnancy and parenting. P‐values indicate differences between agree/completely agree grouped together and disagree/completely disagree grouped together according to training status. **A)** Shows the responses of childbearing participants; **B)** shows the responses of non‐childbearing participants; **C)** shows the agreement in terms of items related to return to work after pregnancy; **D)** shows the general view on the necessity of implementing regulation at the European level on the management of pregnancy during urology residency according to training status.
**Table S4.** Checklist for program directors, combining items, objectives, and corresponding questions for effective discussions with residents.
