# Supplementary material for: Barriers to pregnancy and parenthood during urology residency across Europe
Source: BJUI Compass. 2026 Mar 31;7(4):e70181. doi: 10.1002/bco2.70181 (PMC13098357; doi:10.1002/bco2.70181)
Supplement: Supplementary file 1 — Data S1. Supporting Information [file BCO2-7-e70181-s002.pdf]

European Society of Residents in Urology

ESRU survey on pregnancy and parenting in Europe: for residents

1. Introduction

The European Society of Residents in Urology (ESRU) is conducting research on working regulations on pregnancy and parenting for residents in urology across European countries. We aim to gather your perspectives and offer recommendations for standardized European guidelines.

Target: Residents in urology. If you are a young consultant or fellow, use the following link https://www.surveymonkey.com/r/pregnancyurologists.

The survey will take 10 minutes of your time.

Your responses will be treated confidentially and only for research purposes. By taking part in the survey, you are giving your informed consent to analyze your responses.

European Society of Residents in Urology

ESRU survey on pregnancy and parenting in Europe: for residents

2. Section A: Demographics

* 1. What is your age?

* 2. How do you identify yourself?

- ◯ Female
- ◯ Male
- ◯ Non-binary
- ◯ Prefer not to answer/not listed

* 3. In which country are you pursuing your residency in urology?

* 4. In which year of urology residency training are you currently?
Note: this **includes** years spent in other specialties/departments.

* 5. **During the last month, on average,** how many **hours per week** did you **actually** work (including study hours for your residency)?

* 6. In what type of facility are you accomplishing **most** of your residency training?

- ○ University hospital (i.e. associated with a medical school, with urology residency)
- ○ Non-university hospital (i.e. not associated with a medical school, with urology residency)
- ○ Non-academic hospital (i.e. community hospital, without medical school and without urology residency)

* 7. Which of the following **best** represents your career direction?

- ○ Hospital, academic track (combining clinical/surgical practice with teaching/research)
- ○ Hospital, non-academic track (focusing on clinical/surgical practice)
- ○ Private practice
- ○ Other (such as: industry, administration, research)

* 8. In which urology subspecialty are you **mainly** interested in for your career?

* 9. Which of the following best describes your current relationship status?

- ○ Single (not married)
- ○ Single (separated/divorced)
- ○ Single (widowed)
- ○ Married
- ○ In a relationship (including engagement)

esru
European Society of Residents in Urology

ESRU survey on pregnancy and parenting in Europe: for residents

3. Partner/Spouse

* 10. If you have a partner/spouse, which of the following best describes your relationship setting?
* relationship in the same city, but in a different household
** relationship across different cities or countries

- ◯ Living in the same household
- ◯ Short-distance relationship*
- ◯ Long-distance relationship**

* 11. Which of the following best describes the professional position of **your partner**?

- ◯ Employed but not as a physician or resident
- ◯ Specialized physician or resident in a medical field
- ◯ Specialized physician or resident in a surgical field
- ◯ Not employed

* 12. **During the last month, on average,** how many **hours per week** did **your partner actually** work?

esru
European Society of Residents in Urology

ESRU survey on pregnancy and parenting in Europe: for residents

4. Section B: Family goals

* 13. Do you have children?

- ◯ No
- ◯ Yes, I have 1 child
- ◯ Yes, I have 2 children
- ◯ Yes, I have 3 or more children

esru
European Society of Residents in Urology

ESRU survey on pregnancy and parenting in Europe: for residents

5. Section B: Family goals

NB: if you feel that the question/s does/do not apply to you, select N/A

* 14. At which stage of your career did you have a child/children?

- ◯ Before starting residency
- ◯ During residency
- ◯ In both circumstances

* 15. If you had a child during residency, was it **during a break from residency** or **did you have to take a break from residency**?

- ◯ No
- ◯ Yes, it was time off / I took time off from residency (sabatical)
- ◯ Yes, I was doing research/PhD/Masters
- ◯ N/A
- ◯ None of the above

* 16. During pregnancy, on **average**, how many **hours per week** did you work?

- ◯ ≤30 hours
- ◯ 31-40 hours
- ◯ 41-50 hours
- ◯ 51-60 hours
- ◯ 61-70 hours
- ◯ 70+ hours
- ◯ N/A

* 17. Did you reduce your working schedule during **your/your partner's pregnancy**?

- ◯ No
- ◯ Yes

If yes, please specify at how many weeks of gestation

____

* 18. If not, why?

- ☐ I did not want to be considered weak by my colleagues (**stigma**)
- ☐ I was concerned about financial loss (**econonomic reason**)
- ☐ I was concerned with burdening my colleagues with extrawork (**guilt**)
- ☐ I did not want to miss training or career opportunities
- ☐ I wanted to, but my work could not accomodate it
- ☐ I did not feel like I needed to
- ☐ N/A
- ☐ None of the above

* 19. Please rate how strongly you agree or disagree with the following statements:
"During pregnancy, I felt…

	Strongly disagree	Disagree	Neutral	Agree	Strongly agree	N/A
**stigma or resentment from colleagues** by reducing my work/OR/call hours	○	○	○	○	○	○
**guilty about burdening my colleagues** by reducing my work/OR/call hours	○	○	○	○	○	○
that I **missed *training* opportunities** (i.e., reduced surgical exposure, reduced learning, …)	○	○	○	○	○	○
that I **missed *career* opportunities** (i.e., promotions, …)	○	○	○	○	○	○
that I **could fall behind in training** compared to my peers	○	○	○	○	○	○
that I **could lose my job**	○	○	○	○	○	○
that my colleagues/supervisors were **not** supportive of me	○	○	○	○	○	○

* 20. Did you reduce your working schedule *after* **maternity/paternity leave**?

○ No

○ Yes

If yes, please specify at how many weeks of gestation

* 21. If not, why?

☐ I did not want to be considered weak by my colleagues (**stigma**)

☐ I was concerned about financial loss (**economic reason**)

☐ I was concerned with burdening my colleagues with extrawork (**guilt**)

☐ I did not want to miss training or career opportunities

☐ I wanted to, but my work could not accomodate it

☐ I did not feel like I needed to

☐ N/A

☐ None of the above

ESRU survey on pregnancy and parenting in Europe: for residents

6. Section B: Family goals

NB: if you feel that the question/s does/do not apply to you, select N/A

* 22. Please rate how strongly you agree or disagree with the following statements:
"I am concerned that, If I had a child during residency,...

	Strongly disagree	Disagree	Neutral	Agree	Strongly agree	N/A
... my working conditions would be **unsafe** for my health"	○	○	○	○	○	○
... my working conditions would be **unsafe** for the health of my child"	○	○	○	○	○	○
... I might miss ***training*** opportunities (i.e., reduced surgical exposure, reduced learning, ...)"	○	○	○	○	○	○
... I might miss ***career*** opportunities (i.e., promotions, ...)"	○	○	○	○	○	○
... I might **fall behind in training** compared to my peers"	○	○	○	○	○	○
... I might lose my job"	○	○	○	○	○	○
... I would **burden my colleagues with additional working/call hours**"	○	○	○	○	○	○
... my colleagues/supervisors would **not** be supportive of me"	○	○	○	○	○	○
...my job would **not** give me enough time to spend with my child"	○	○	○	○	○	○

ESRU survey on pregnancy and parenting in Europe: for residents

7. Section C: Pregnancy regulations

Regulations in your urology department/division (internal policies).

* 23. Is there a written policy describing how pregnancy-related aspects (leave, training adaptation upon return to work, etc.) during residency ***should*** be managed in your department?

- ◯ Yes
- ◯ No
- ◯ I don’t know

* 24. At the beginning of your residency, **was it discussed** with your boss or one of your supervisors how **pregnancy** *and/or* **parental leave** during residency is managed in your department, **or not**?

- ◯ Yes
- ◯ No
- ◯ I don’t remember

* 25. If not, do you think it would have been **important or not** to have had such a conversation?

- ◯ Very important
- ◯ Quite important
- ◯ Neutral
- ◯ Quite unimportant
- ◯ Very unimportant

esru

European Society of Residents in Urology

ESRU survey on pregnancy and parenting in Europe: for residents

8. Section C: Pregnancy regulations

National regulations/recommendations (according to constitution/medical association/urological association)

NB: "your country" = the country you are pursuing your residency.

* 26. What is the regulation concerning exposure* to ionizing radiations during pregnancy within urology residency *in your country*?
* any activity requiring the use of ionizing radiations, with the pregnant person working within a few meters from the radiation source

- Exposure *is* permitted
- Exposure should *be* avoided
- Exposure must *be* avoided
- There is no official regulation
- I don’t know

* 27. What is the regulation concerning participation to **surgical procedures** (as operator or assistant) during pregnancy within urology residency *in your country*?

- It is *prohibited* **to participate** in *all* surgical procedures
- It is *prohibited* **to participate** in *some* surgical procedures
- It is *recommended* to **avoid participation** in *all* surgical procedures
- It is *recommended* to **avoid participation** in *some* surgical procedures
- It is *allowed* to *participate to any* surgical procedure
- There is no official regulation
- I don’t know

* 28. *Your opinion:* What do you think about the participation to **surgical procedures** (as operator or assistant) during pregnancy within urology residency?

- It *should **not*** be allowed to access to any surgical procedures
- It *should **not*** be allowed to access to some surgical procedures (such as long surgeries or surgeries requiring radiation exposure)
- It *should* be allowed to participate to any surgical procedure
- I don’t know

esru
European Society of Residents in Urology

ESRU survey on pregnancy and parenting in Europe: for residents

9. Maternity and paternity leave

National regulations/recommendations (according to constitution/medical association/urological association)

NB: "your country" = the country you are pursuing your residency.

* 29. What is total duration of standard *paid* (full and reduced) maternity leave (*before* and after *birth*) within urology residency *in your country*?

* 30. What is the total duration of *unpaid* maternity leave within urology residency *in your country*?

* 31. Does maternity leave extend the duration of urology residency *in your country*?

- No
- Yes
- It depends
- I don’t know

* 32. *Your opinion:* Do you think that maternity leave should require an extension of the duration of urology residency?

- No
- Yes
- It depends
- I don’t know

* 33. What is the duration of paid paternity leave within urology residency *in your country*?

* 34. Does taking paternity leave require extending the duration of urology residency *in your country*?

- No
- Yes
- It depends
- I don’t know

* 35. *Your opinion:* Do you think that taking paternity leave should require an extension of the duration of urology residency?

- No
- Yes
- It depends
- I don’t know

* 36. Do you **agree or disagree** that having the possibility to **split the parental leave** would be an advantage for a family?

- ◯ Strongly agree
- ◯ Agree
- ◯ Neither agree nor disagree
- ◯ Disagree
- ◯ Strongly disagree

European Society of Residents in Urology

ESRU survey on pregnancy and parenting in Europe: for residents

10. Return to work: adaptation of working schedule, lactation, working environment

National regulations/recommendations (according to constitution/medical association/urological association)

NB: "your country" = the country you are pursuing your residency.

* 37. Is there any **regulation** concerning the **adaptation of the *working* schedule** (e.g. reduction to a part-time working) upon return to work *in your country*?

- ◯ Yes, there is an official regulation
- ◯ No, there is no official regulation
- ◯ I don’t know

* 38. Is there any **regulation** regarding the **adaptation of the *training* schedule** to achieve planned urology residency objectives upon return to work *in your country*?

- ◯ Yes, there is an official regulation
- ◯ No, there is no official regulation
- ◯ I don’t know

* 39. Is the frequency of **breastfeeding breaks** during working hours legally regulated *in your country*?

- ◯ Yes, it is legally regulated
- ◯ No, it is not legally regulated
- ◯ I don’t know

* 40. Are employers required to provide a **private lactation space** in proximity to your work environment?

- ◯ Yes
- ◯ No
- ◯ I don’t know

* 41. Are employers required to **facilitate the working environment** towards access to childcare *in your country*?

- Yes
- No
- I don't know

* 42. *Your opinion*: Do you **agree or disagree** that...

	Strongly disagree	Disagree	Neutral	Agree	Strongly agree	N/A
...the ***working*** **schedule** should be **adapted** (e.g. reduction to a part-time working) **upon return to work after pregnancy**, if requested by the trainee?						
...the ***training*** **schedule** should be **adapted to achieve planned urology residency objective** upon return to work after pregnancy?						
...there should be **protected time for breastfeeding** during working hours?						
...a **private lactation space** should be provided in proximitity to your work environment?						
...employers should be required to facilitate the working environment towards **access to childcare**?						

esru

European Society of Residents in Urology

ESRU survey on pregnancy and parenting in Europe: for residents

11. Final questions

* 43. Recommendations on which of these topics do you **agree or disagree** should be **included** in the framework of **general European recommendations** about the management of pregnancy during urology residency?

	Strongly disagree	Disagree	Neutral	Agree	Strongly agree	N/A
Duration of maternal leave during pregnancy	○	○	○	○	○	○
Night work during pregnancy	○	○	○	○	○	○
Working on holidays during pregnancy	○	○	○	○	○	○
Exposure to ionizing radiations during pregnancy	○	○	○	○	○	○
Duration of maternal leave after pregnancy	○	○	○	○	○	○
Duration of paternal leave after pregnancy	○	○	○	○	○	○
Necessity to extend the duration of the residency program	○	○	○	○	○	○
Adaptation of the working schedule upon return to work after pregnancy	○	○	○	○	○	○
Adaptation of the training schedule upon return to work after pregnancy	○	○	○	○	○	○
Frequency of breastfeeding	○	○	○	○	○	○
Access to a lactation space	○	○	○	○	○	○
Facilitated access to childcare	○	○	○	○	○	○
Providing written internal guidelines on the management of pregnancy	○	○	○	○	○	○
Suggesting open discussion of the institutional management of pregnancy at the beginning of residency	○	○	○	○	○	○

44. Do you have any other questions, comments or opinions on this issue? If yes, please write them below.

European Society of Residents in Urology

ESRU survey on pregnancy and parenting in Europe: for residents

12. Congratulations on completing the survey!

We will let you know about the results soon.

In the meanwhile, please share the survey with your colleagues!
