# Supplementary material for: Barriers to pregnancy and parenthood during urology residency across Europe
Source: BJUI Compass. 2026 Mar 31;7(4):e70181. doi: 10.1002/bco2.70181 (PMC13098357; doi:10.1002/bco2.70181)
Supplement: Supplementary file 3 — Figure S1. Pie chart illustrating the importance for residents to discuss with their boss or supervisor how pregnancy/parenting are managed in their department. Table S1. European Country‐Level legal protections on pregnancy and parenthood. Hereby we present a list of maternity leave, paternity leave, parental leave, radiation protection for pregnant workers, workplace accommodations (lactation rooms, breastfeeding breaks, childcare), and post‐leave schedule/residency rules for most European countries. Medical training/residency context is prioritized where applicable. Data reflect current frameworks circa 2024–2025; see sources in the last column for each country. Table S2. Sociodemographic data of respondents after stratification by training status. Table S3. Internal regulations regarding management of pregnancy during pregnancy after stratification by training status. Figure S2. Diverging stacked bar plot illustrating the views of residents and consultants in urology on different topics related to pregnancy and parenting. P‐values indicate differences between agree/completely agree grouped together and disagree/completely disagree grouped together according to training status. A) Shows the responses of childbearing participants; B) shows the responses of non‐childbearing participants; C) shows the agreement in terms of items related to return to work after pregnancy; D) shows the general view on the necessity of implementing regulation at the European level on the management of pregnancy during urology residency according to training status. Table S4. Checklist for program directors, combining items, objectives, and corresponding questions for effective discussions with residents. [file BCO2-7-e70181-s001.docx]

**Supplementary Figure 1**. Pie chart illustrating the importance for residents to discuss with their boss or supervisor how pregnancy/parenting are managed in their department.


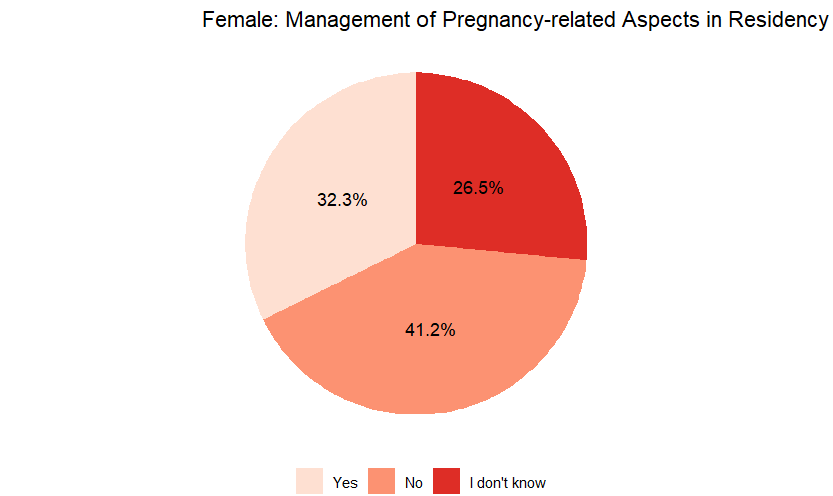

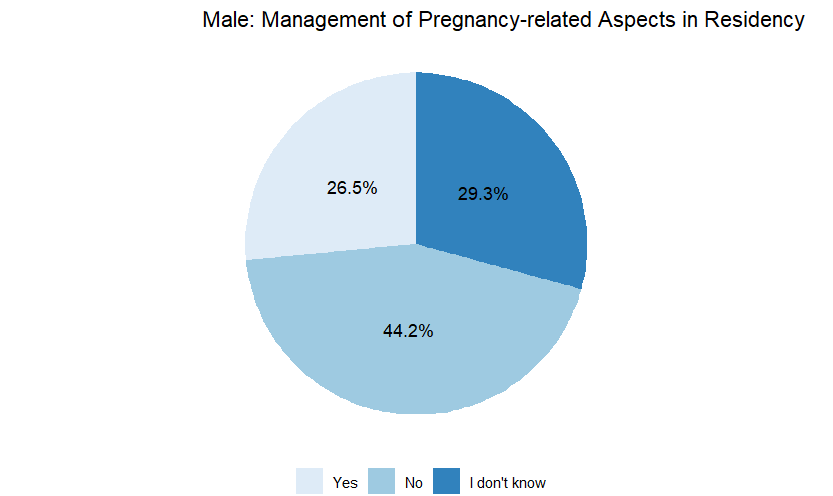


*P-value: 0.4*


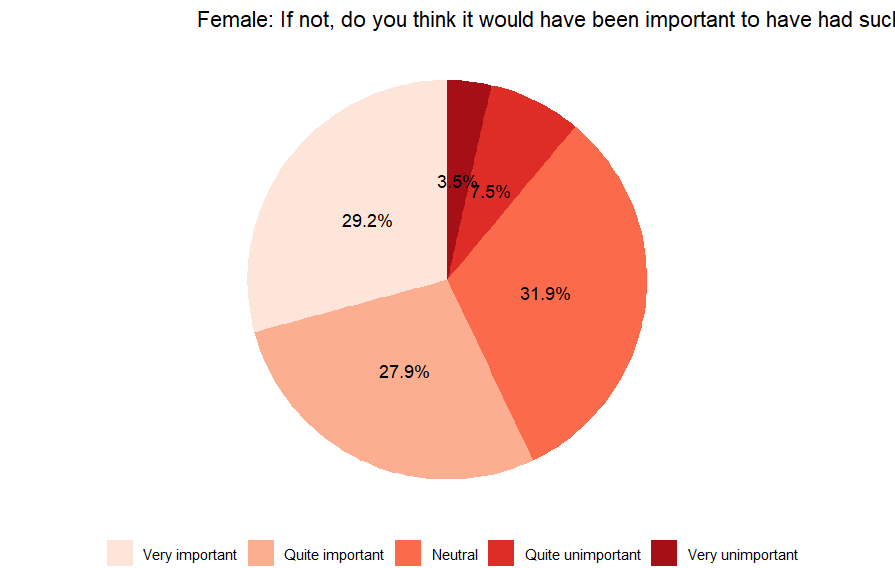

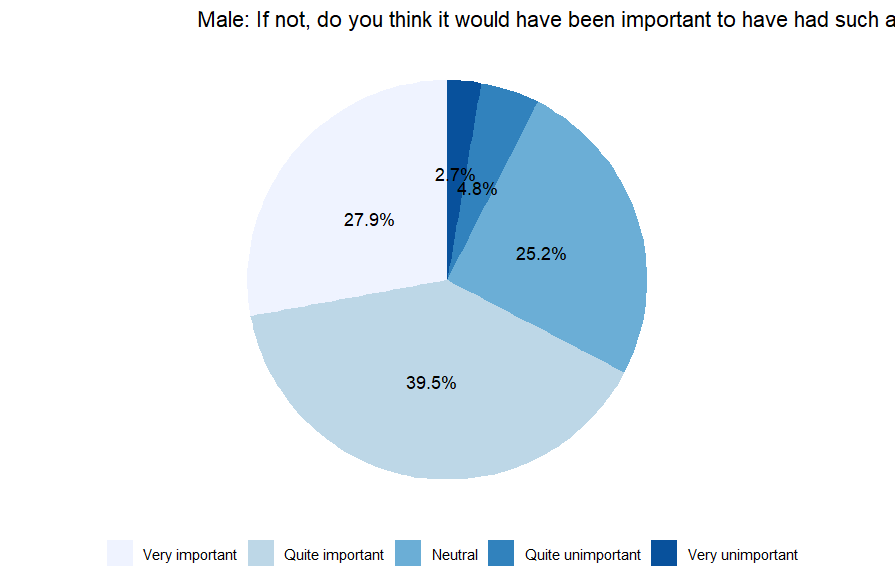


*P-value: 0.1*


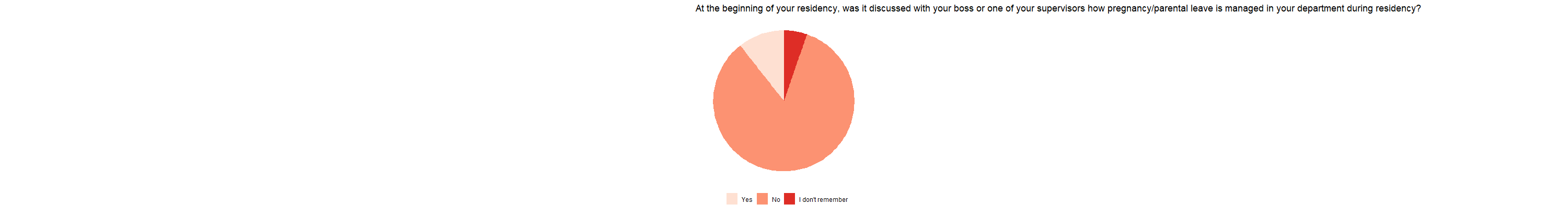

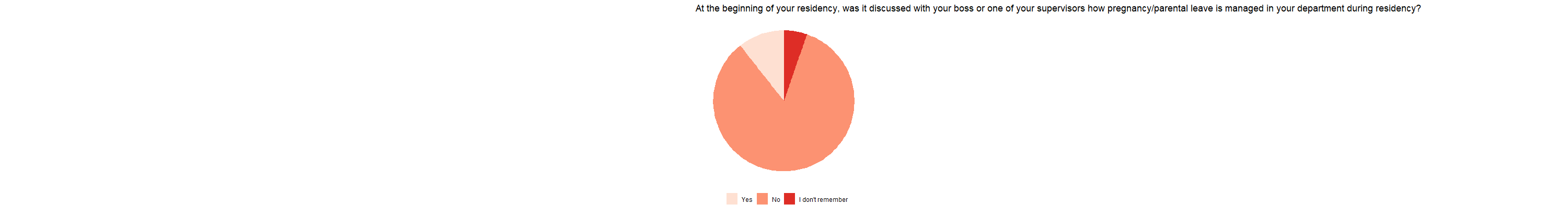

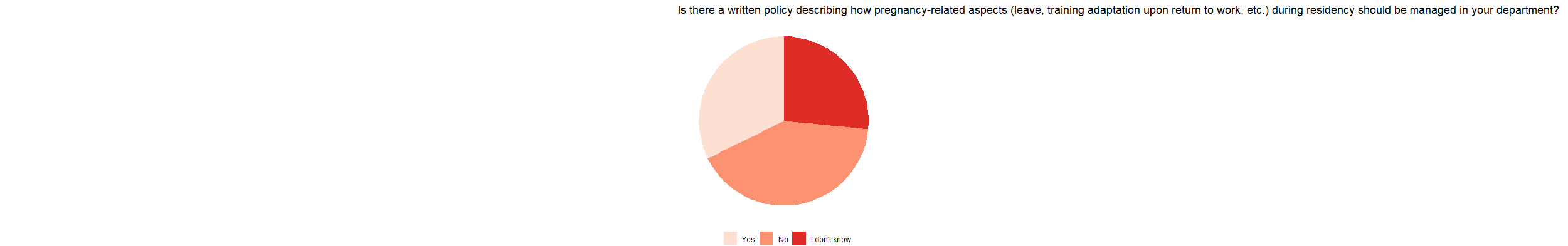

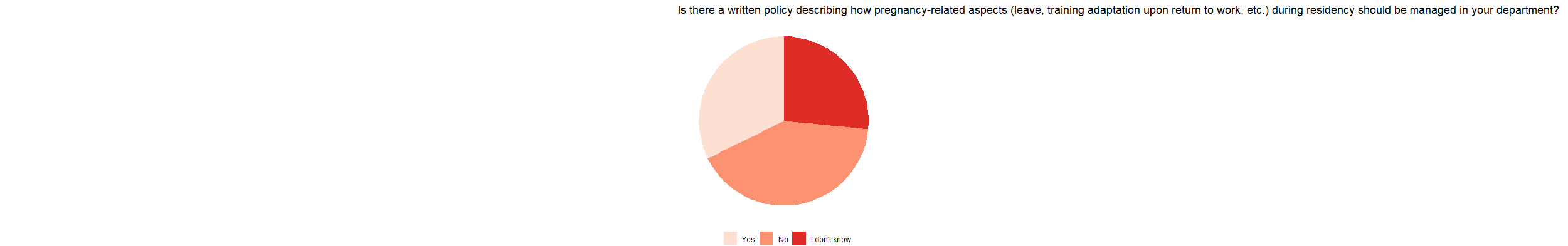


**Males**

**Females**

*P-value: 0.5*


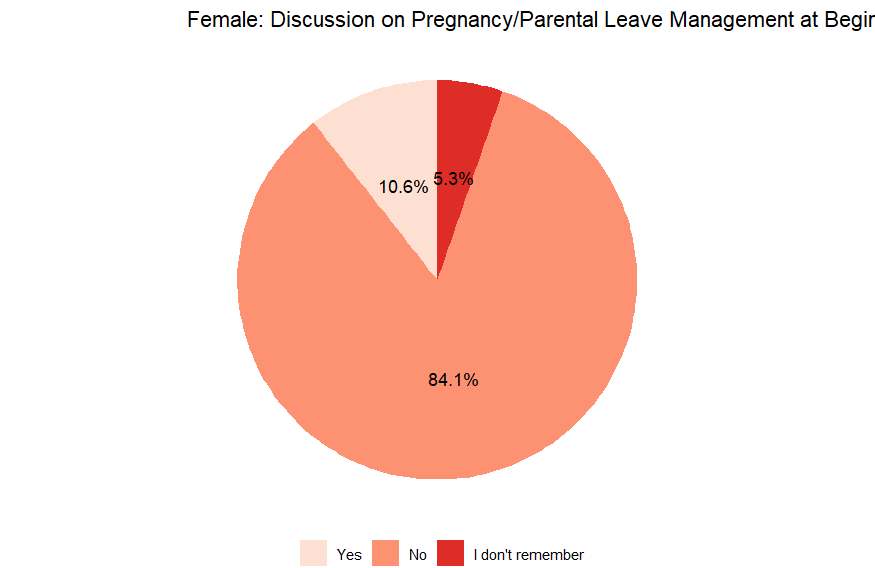

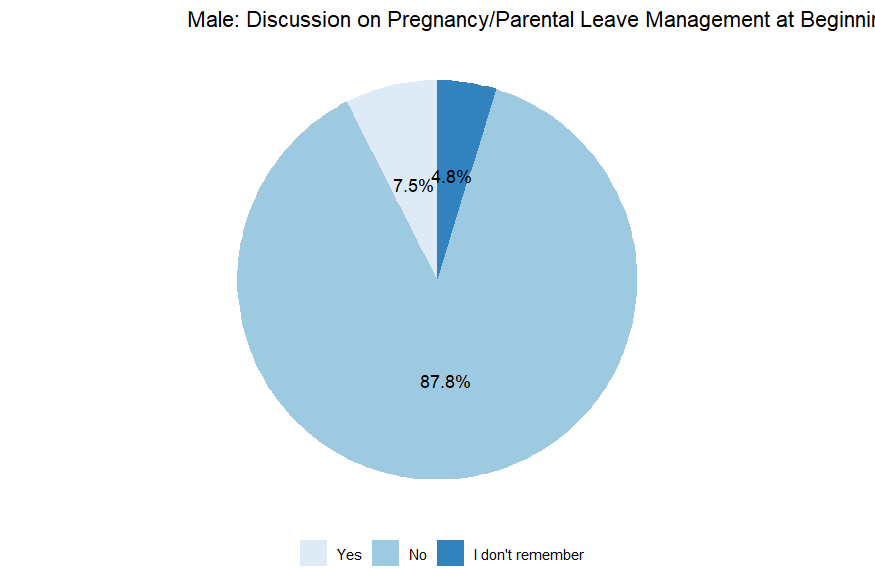

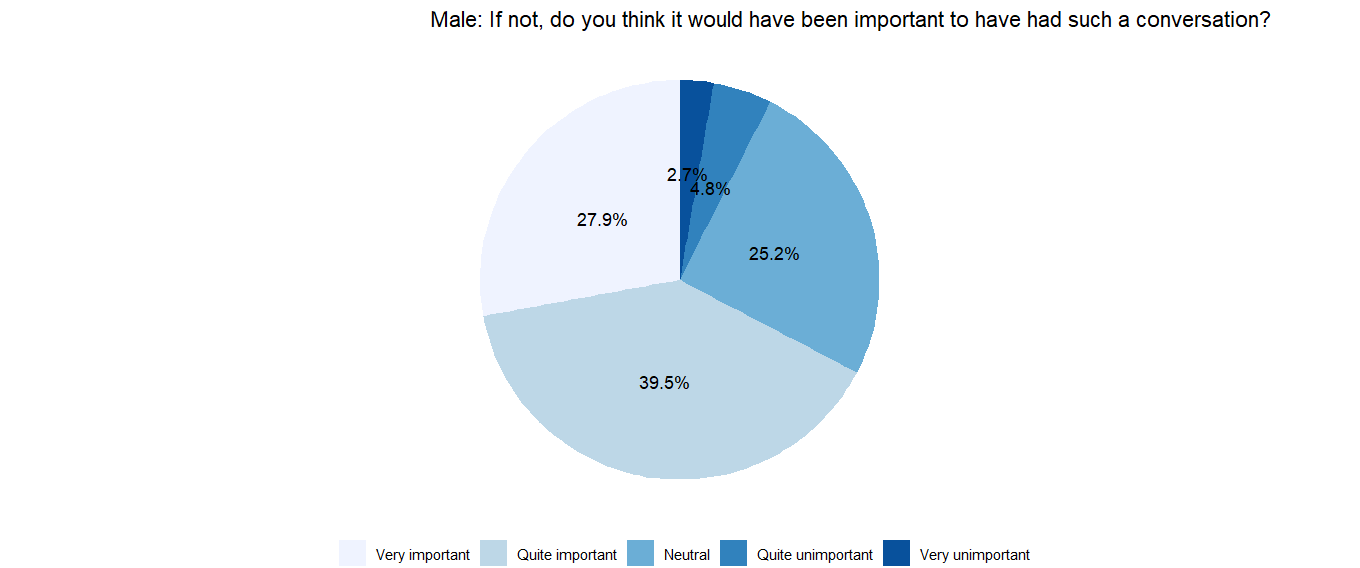


**Supplementary Table 1**: European Country-Level legal protections on pregnancy and parenthood. Hereby we present a list of maternity leave, paternity leave, parental leave, radiation protection for pregnant workers, workplace accommodations (lactation rooms, breastfeeding breaks, childcare), and post-leave schedule/residency rules for most European countries. Medical training/residency context is prioritized where applicable. Data reflect current frameworks circa 2024-2025; see sources in the last column for each country.

| Country / Jurisdiction | Maternity Leave | Paternity / Partner Leave | Parental Leave | Radiological Exposure (Pregnant Workers) | Lactation Rooms (workplace mandate) | Breastfeeding Breaks | Childcare / Employer Support | Post-Leave Schedule / Residency Rules | Key Sources |
| --- | --- | --- | --- | --- | --- | --- | --- | --- | --- |
| European Union (EU Directives) | Minimum 14 weeks maternity leave; at least 2 weeks compulsory; pay at least equivalent to sick pay (country decides exact rate). | Minimum 10 working days paternity leave around birth; paid at least at sick-pay level. | At least 4 months per parent, 2 months non‑transferable; right to request flexible uptake (part‑time/blocks); non‑transferable portion should be adequately compensated (set nationally). | Pregnant workers must be protected from hazards; once notified, fetus must be protected as a member of the public; fetal dose kept ALARA and unlikely to exceed 1 mSv remainder of pregnancy. | Risk assessment and reasonable accommodations required; EU does not mandate lactation rooms, but Member States may impose stricter rules. | Member States set specific rules; EU does not fix paid nursing breaks, but general protection for breastfeeding workers applies under Pregnant Workers Directive. | No EU mandate for employer childcare; Member States handle childcare policy. Anti-discrimination protections and right to return to same/equivalent post after leave. | No EU-wide residency rules; medical training organized nationally. EU encourages non-discrimination and work-life balance; national boards set reintegration policies. | Directive 92/85/EEC (Pregnant Workers); Directive (EU) 2019/1158 (Work–Life Balance); Council Directive 96/29/Euratom & 2013/59/Euratom (radiation). |
| Austria | 16 weeks (8 pre/8 post) ~100% via social insurance. | 4 weeks unpaid job-protected "Papamonat"; lump-sum state benefit. | Parental leave up to child’s 2nd birthday; split between parents; unpaid (various childcare benefit options). | Reassignment away from ionizing radiation; fetal dose ≤1 mSv; employer must offer alternative duties. | No general mandate for lactation rooms; employers must provide rest facilities for pregnant workers. | No explicit statutory nursing breaks beyond EU minimum; flexible arrangements common; part-time (Elternteilzeit) widely used. | No employer childcare mandate (public childcare and allowances available). | No residency-specific rule; right to part-time until child age 7; breastfeeding mothers protected from night/overtime by medical certificate. | EU 92/85/EEC; Austrian Mutterschutz rules; national radiation code. |
| Belgium | 15 weeks (6 pre + 9 post; 9 post compulsory); 82% first 30 days then 75% (capped). | 20 days birth/partner leave (3 days 100% employer + 17 days 82% social security). | 4 months per parent; can take part-time formats; unpaid with state allowance; non-transferable. | Exposure allowed only if ≤1 mSv fetal dose and risk analysis with radiation expert; otherwise reassignment. | Yes, for larger employers (≥100 female employees) dedicated nursing room required; smaller employers must still allow private space. | Paid nursing breaks up to 9 months postpartum (e.g., 1 hour per day if ≥7.5 h work), reimbursed via health insurance. | No employer childcare mandate (public nursery network; voluntary company crèches). | No residency-specific national rules; general protections (no night/overtime if unsafe), flexible/part-time upon return by agreement. | Belgian social security portal; EU 2013/59/Euratom; national employment code on lactation rooms. |
| Bulgaria | 410 days (~58 weeks) at ~90% pay (social insurance). | 15 days paid at ~90% (social insurance). | Unpaid childcare leave till age 2 with flat benefit; each parent 6 months parental leave (50% minimum wage) until age 8; some portions non‑transferable | Prohibits hazardous work incl. ionizing radiation; reassignment or leave required. | Required mother-and-baby rooms at larger employers (thresholds e.g., ≥50 women). | Two 30‑min paid breaks daily (or 1 hour) until 8–12 months; can combine/shift by agreement. | No employer childcare duty; public kindergarten places; some tax incentives. | No residency-specific rules; breastfeeding mothers protected from night/overtime; strong dismissal protections for parents. | Bulgarian Labour Code; national OSH rules for pregnant/breastfeeding workers. |
| Croatia | Mandatory maternity ~6 months post-birth at 100% (no ceiling); additional maternity to child’s 1st birthday (capped). | 10 working days paid at 100% (capped). | Each parent 8 months (if both use, total 16 months), until child 8; paid flat rates with higher initial amounts. | Reallocation from hazardous duties; avoid radiation exposure; risk assessment and paid leave if needed. | No specific mandate for lactation rooms (best practice in hospitals). | Two × 60‑min paid nursing breaks (full-time) to 1 year; extendable on pediatric advice. | No employer childcare mandate; public daycare available. | No residency-specific national rule; night/overtime restricted for breastfeeding mothers; flexible/part-time by agreement. | Croatian Labour Act; OSH pregnancy protection rules. |
| Cyprus | 22 weeks (~5 months) ~72% pay (social insurance); 11 weeks compulsory. | 2 weeks (~10 working days) ~72% pay. | 18 weeks unpaid parental leave per parent (up to 8 weeks/year) to child age 8; new limited paid portions introduced. | Fetal dose ≤1 mSv; reassignment away from ionizing radiation if risk can’t be controlled. | No statutory mandate for lactation rooms; reasonable accommodations expected. | 1 hour/day nursing until 9 months (generally unpaid unless agreed/covered). | No employer childcare duty; right to request flexible work recently transposed. | No residency-specific rules; flexible work requests allowed; schedule easing at employer discretion. | Cyprus social insurance; EU transposition notes; national OSH law. |
| Czechia | 28 weeks (single birth) at ~70% pay; 37 weeks for multiples. | 2 weeks at ~70% pay. | Parental leave until age 3 (job-protected) with parental benefit (family-level, flexible duration). | Pregnant workers not allowed in Category A radiation; fetal dose ≤1 mSv; reassignment required. | No statutory lactation-room mandate (private space commonly provided). | Two 30‑min breaks (practice varies on pay); part‑time often arranged. | No employer childcare duty; state supports daycare; company kindergartens voluntary. | No residency-specific rules; right to reduced hours until child 15; night/overtime limits for breastfeeding/ single parents of infants. | Czech Labour Code; social benefits act; radiation safety decree. |
| Spain | 16 weeks at 100% (6 weeks compulsory post‑birth). | 16 weeks at 100% (equal to maternity). | Unpaid parental leave to child age 3 (job-protected). New 8 weeks additional parental leave per parent (at least 2 weeks paid since 2024). | Fetal dose ≤1 mSv; pregnant residents reallocated away from X‑ray/fluoro; employer must modify tasks. | Employers must provide suitable private lactation space (equality and OSH obligations). | Paid ‘lactation hour’: 1 hour/day (two × 30 min or reduction of day) to 9–12 months, with pay; can accumulate by agreement. | No employer childcare duty; strong right to request reduced hours until child 12. | Residency generally extended by leave taken; surveys report ~58% needing extension for maternity leave; flexible/part‑time residency not standard. | BOE Estatuto de los Trabajadores; equality law on breastfeeding; Social Security; trainee survey (ES). |
| Italy | 5 months (2+3 or 1+4) at 80%. | 10 working days at 100% (mandatory). | Each parent up to 6 months; 9 months paid at 30% total (recently expanded) + 1 month at 80% if within first 6 years. | Reassignment away from ionizing radiation per DLgs 151/2001; fetal dose ≤1 mSv. | Employers must set up a suitable nursing area/room. | Two paid nursing breaks (total 2 hours/day full‑time) to 1 year; fathers eligible if mother not employed. | No employer childcare duty; right to request flexible/part‑time; prioritization for parents. | Residency: >40 consecutive days absence suspends training; maternity leave time added to program; part‑time training possible. | DLgs 151/2001; INPS circulars; specialty training rules (MIUR/Universities). |
| France | 16 weeks (6 pre + 10 post, longer for later births) ~ 90-100% up to ceiling (SS indemnity). | 28 days (3 employer + 25 SS) paid. | Parental leave (congé parental) up to 3 years per child (unpaid by employer; CAF flat benefit). | Pregnant staff barred from Category A ionizing radiation and other high-risk tasks; fetal dose kept ≤1 mSv. | Labour inspectors can require nursing rooms in workplaces with ≥100 women; all employers must enable a clean private space. | 1 hour/day for first year (2×30 min), generally unpaid unless CBA provides; many hospitals accommodate. | No employer childcare duty; extensive public crèches; many employers offer nursery places voluntarily. | Residency time is extended by the length of leave to meet rotation/time requirements; return to next rotation; no penalty beyond delay. | Code du Travail (L1225‑30 ff.); Assurance Maladie; INRS radiation guidance. |
| Germany | 14 weeks (6 pre + 8 post; 12 post for multiple/ premature) at 100% net (Mutterschaftsgeld + employer top‑up). | 2 weeks partner leave. | Elternzeit up to 3 years per parent (job-protected); Elterngeld ~65% for 12-14 months total. | Pregnant workers removed from significant radiation; fetal dose ≤1 mSv; reassignment mandatory. | Suitable private room must be provided; breastfeeding/ pumping accommodations required. | Paid nursing breaks: ≥60 min/day up to 12 months (e.g., 2×30 min); cannot be offset against rest breaks. | No employer childcare duty; guaranteed daycare from age 1; flexible/part-time rights strong. | Residency: time-based; leaves extend training; part‑time training widely used (50-80%); discrimination for taking leave prohibited. | Mutterschutzgesetz; BfS radiation rules; Elterngeld/Elternzeit law. |
| United Kingdom | Up to 52 weeks leave; 39 weeks paid (6 weeks 90% pay + 33 weeks at flat rate or 90% if lower). | 1-2 weeks at statutory paternity pay. | Shared Parental Leave up to 50 weeks leave/ 37 weeks pay shared; also 18 weeks unpaid parental leave per parent (until child 18). | Risk assessment; remove from high-exposure duties; fetal dose <1 mSv guided by IRR; NHS policies accommodate pregnant staff in radiology/theatre. | Employers must provide a place to rest and a private space to express milk (not a bathroom). | No statutory paid nursing breaks; employers expected to be flexible (NHS guidance supports). | No employer childcare duty; Tax‑Free Childcare & workplace nurseries optional; universal right to request flexible working. | Residency: >14 days absence in training year extends program; national SuppoRTT scheme for return; LTFT training widely available. | NHS Employers; BMA; UK gov maternity/paternity guidance; HSE/IRR radiation rules. |
| Denmark | 18 weeks mother (4 pre + 14 post) with benefits (capped). | 2 weeks father/ partner paid. | 32 weeks shared; 11 weeks earmarked to each parent paid; remaining transferable. Total ~52 weeks paid/ unpaid mix. | Remove from high-radiation duties; fetal dose ≤1 mSv; hospital policies ensure reassignment. | No explicit lactation-room statute; private, safe space expected; night/overtime limits if harmful. | Breastfeeding breaks accommodated by practice; many mothers remain on leave during nursing. | No employer childcare duty; universal public childcare; flexible hours common. | Residency pauses during leave; end date shifts; part‑time training common and supported. | Denmark Leave Act (2022 reform); Working Environment Act; hospital HR policies. |
| Netherlands | 16 weeks at 100% (4-6 pre + 10–12 post). | 1 week 100% + 5 weeks at 70% within 6 months of birth. | 26 weeks parental leave per parent to age 8; first 9 weeks at ~70% if taken in first year; remainder unpaid. | Remove from Category A radiation; fetal dose ≤1 mSv; adjust duties and monitor dosimetry. | Must provide private comfortable room (not a toilet) for nursing/pumping. | Up to 25% of working time for breastfeeding/pumping with full pay for first 9 months. | No employer childcare duty; generous state childcare allowance; some on‑site nurseries voluntarily. | Residency: leave pauses training; part‑time specialty training common (e.g., 80%); completion date adjusts to meet competencies. | UWV/Overheid.nl leave rules; Arbeidstijdenwet breastfeeding rights; hospital radiation safety. |
| Portugal | Initial parental leave 120 days at 100% (or 150 days at 80%); mandatory 6 weeks for mother post‑birth. | 28 days mandatory (7 immediately post‑birth), paid; bonus days if both parents share. | Additional unpaid parental leave to child’s 3rd birthday; options for partial-paid part-time leave after regular leave. | Reassignment from ionizing radiation; fetal dose ≤1 mSv; OSH law protects pregnant workers. | Employers must allow nursing schedule/space; no universal room mandate but common in hospitals. | Two paid daily breaks (~1 hour each) until child’s 1st birthday; extendable if breastfeeding continues. | No employer childcare duty; public/private daycare with subsidies; flexible work rights for parents. | Residency: training paused; end date extended by leave; individualized support to complete rotations/exams. | Portuguese Labour Code; Social Security; OSH pregnancy regulations. |
| Switzerland | 14 weeks (98 days) at 80% pay (federal); some cantons/employers top-up. | 2 weeks (10 working days) at 80% pay. | No national parental leave; extra leave depends on canton/employer (case-by-case). | Remove from ionizing radiation/harmful exposures; fetal dose ≤1 mSv; paid leave if no safe post available. | Employers must provide a suitable private room for nursing; rest facilities required. | Paid breastfeeding time in first year: 30–90 minutes/day depending on length of shift (counted as work time). | No employer childcare duty; many hospitals provide on‑site crèches voluntarily; high part‑time uptake. | Residency: up to 8 weeks per training year can be absent without extension; longer absences extend training; part‑time training widely used. | Swiss Labour Act & OLT 3; EO maternity/paternity insurance; FMH training regulations. |

**Supplementary Table 2**: Sociodemographic data of respondents after stratification by training status.

| **Variables** | **Residents (n=255, 66%)** | **Consultants (n=132, 34%)** | **P-value** |
| --- | --- | --- | --- |
| **Age category**, n (%) |  |  |  |
| <=29 | 81 (31.8) | 1 (0.8) | ***<0.001*** |
| 30-39 | 173 (67.8) | 113 (85.6) |  |
| 40+ | 1 (0.4) | 18 (13.6) |  |
| **Sex**, n (%) |  |  |  |
| Female | 161 (63.1) | 76 (57.6) | 0.3 |
| Male | 94 (36.9) | 56 (42.4) |  |
| **Geographical area of work**, n (%) |  |  |  |
| Southern Europe | 91 (35.7) | 58 (43.9) | ***0.004*** |
| Western Europe | 106 (41.6) | 49 (37.1) |  |
| Northern Europe | 23 (9) | 20 (15.2) |  |
| Central and Eastern Europe | 35 (13.7) | 5 (3.8) |  |
| **Year of residency/consultancy**, n (%) |  |  |  |
| 1-2 | 38 (14.9) | 56 (42.4) | ***<0.001*** |
| 3-5 | 169 (66.3) | 51 (38.6) |  |
| 6-10 | 48 (18.8) | 25 (18.9) |  |
| **Weekly working hours**, n (%) |  |  |  |
| Up to 50 hours | 27 (10.6) | 28 (21.2) | 0.007 |
| More than 50 hours | 198 (77.6) | 92 (69.7) |  |
| N/A | 5 (2) | - |  |
| *Missing* | *25 (9.8)* | *12 (9.1)* |  |
| **Facility type during residency**, n (%) |  |  |  |
| University hospital | 165 (64.7) | 105 (79.5) | 0.007 |
| Non-university hospital | 84 (32.9) | 26 (19.7) |  |
| Non-academic hospital | 6 (2.4) | 1 (0.8) |  |
| **Future (residents) or current (consultant) facility type**, n (%) |  |  |  |
| Hospital, academic track | 103 (40.4) | 90 (68.2) | ***<0.001*** |
| Hospital, non-academic track | 133 (52.2) | 37 (28) |  |
| Private practice | 19 (7.5) | 4 (3) |  |
| Other | - | 1 (0.8) |  |
| **Subspecialty field (aimed at or practiced)**, n (%) |  |  |  |
| General urology | 27 (10.6) | 28 (21.2) | 0.09 |
| Neurourology | 4 (1.6) | 2 (1.5) |  |
| Endourology | 44 (17.3) | 14 (10.6) |  |
| Urologic oncology | 107 (42) | 62 (47) |  |
| Urologic trauma and reconstructive urology | 15 (5.9) | 6 (4.5) |  |
| Pediatric urology | 8 (3.1) | 4 (3) |  |
| Male sexual and reproductive health | 14 (5.5) | 6 (4.5) |  |
| Renal transplantation | 2 (0.8) | 2 (1.5) |  |
| Urogynecology | 6 (2.4) | 6 (4.5) |  |
| Uncertain | 28 (11) | 2 (1.5) |  |
| **Children** | 87 (34.1) | 70 (53) | ***<0.001*** |
| **Parenthood timing** |  |  |  |
| Before residency | 81 (31.8) | 29 (22) | ***<0.001*** |
| During residency | 6 (2.4) | 5 (3.8) |  |
| After residency | - | 36 (27.3) |  |
| No parenthood | 168 (65.9) | 62 (47) |  |

*According to the *United Nations geoscheme*: Southern Europe: Albania, Croatia, Greece, Italy, Macedonia, Portugal, Serbia, Slovenia, Spain, Turkey; Western Europe: Austria, Belgium, France, Germany, The Netherlands, Switzerland; Northern Europe: Denmark, Estonia, Ireland, Latvia, Sweden, United Kingdom; Eastern Europe: Czech Republic, Georgia, Hungary, Poland, Romania, Slovakia.

** Practice Setting: University hospital = associated with a medical school, with urology residency; Non-university hospital = not associated with a medical school, with urology residency; Non-academic hospital = community hospital, without medical school and without urology residency.

*** Future perspective: academic track = combining clinical/surgical practice with teaching/research; non-academic track = focusing on clinical/surgical practice; other = industry, administration, research.

**Supplementary Table 3**: Internal regulations regarding management of pregnancy during pregnancy after stratification by training status.

| Item | Resident (N=255, 66%) | Consultant (N=132, 34%) | P-value |
| --- | --- | --- | --- |
| Presence of a written policy about the management of pregnancy during residency |  |  |  |
| Yes | 71 (27.8) | 41 (31.1) | 0.7 |
| No | 102 (40) | 56 (42.4) |  |
| I don't know | 74 (29) | 29 (22) |  |
| *Missing* | *8 (3.1)* | *6 (4.5)* |  |
| Was it discussed with your boss or one of your supervisors how pregnancy and/or parental leave during residency is managed in your department |  |  |  |
| Yes | 26 (10.2) | 9 (6.8) | 0.8 |
| No | 208 (81.6) | 111 (84.1) |  |
| I don't remember | 13 (5.1) | 6 (4.5) |  |
| *Missing* | *8 (3.1)* | *6 (4.5)* |  |
| If not, do you think it would have been important or not to have had such a conversation? |  |  |  |
| Very important | 66 (25.9) | 41 (31.1) | 0.6 |
| Quite important | 77 (30.2) | 44 (33.3) |  |
| Neutral | 79 (31) | 30 (22.7) |  |
| Quite unimportant | 17 (6.7) | 7 (5.3) |  |
| Very unimportant | 8 (3.1) | 4 (3) |  |
| *Missing* | *8 (3.1)* | *6 (4.5)* |  |

**Supplementary Figure 2**. Diverging stacked bar plot illustrating the views of residents and consultants in urology on different topics related to pregnancy and parenting. P-values indicate differences between agree/completely agree grouped together and disagree/completely disagree grouped together according to training status. **A)** Shows the responses of childbearing participants; **B)** shows the responses of non-childbearing participants; **C)** shows the agreement in terms of items related to return to work after pregnancy; **D)** shows the general view on the necessity of implementing regulation at the European level on the management of pregnancy during urology residency according to training status.

**
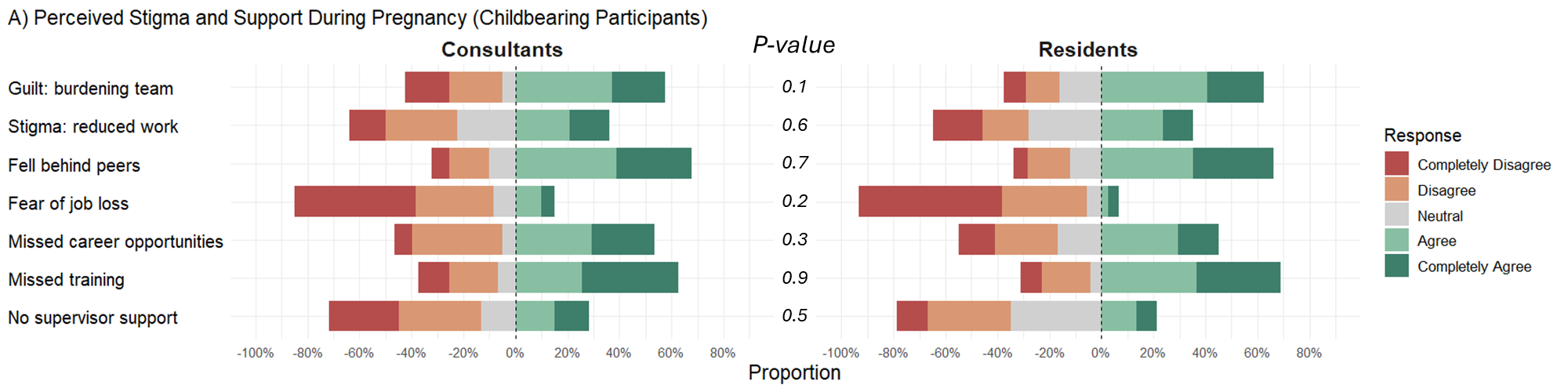
**

**
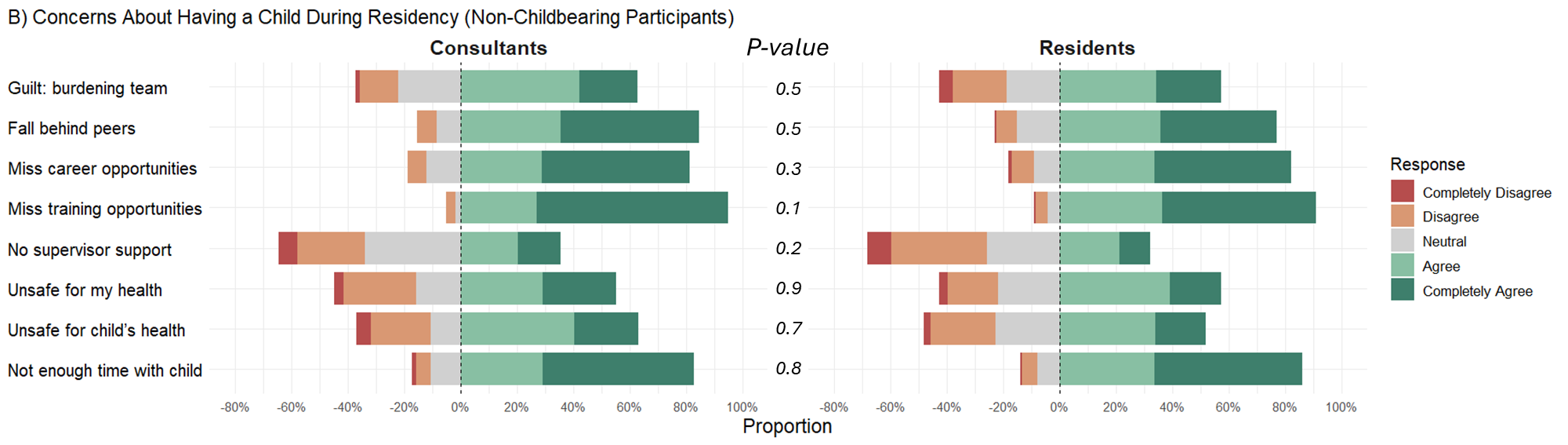
**

**
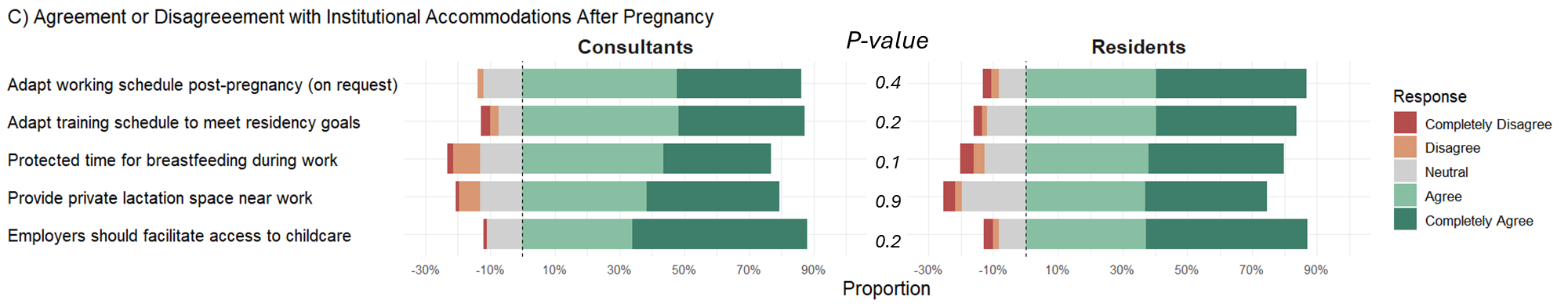
**

**
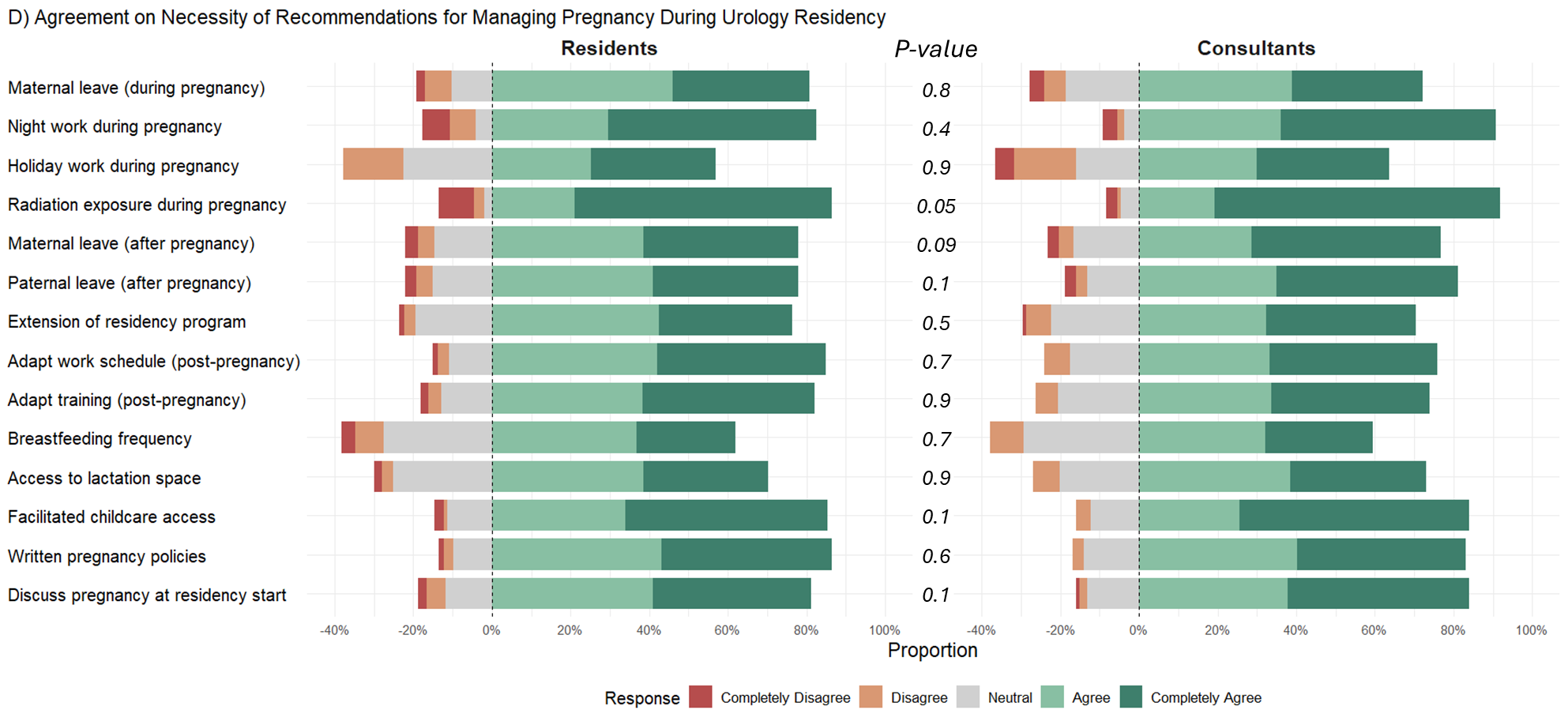
**

**Supplementary Table 4**. Checklist for program directors, combining items, objectives, and corresponding questions for effective discussions with residents.

| Item | Purpose/Objective | Question |
| --- | --- | --- |
| Planning a family during residency | To establish a clear understanding of institutional support and policies. | Would you like to know our department policy about the management of pregnancy and parenthood during residency training? |
| Training schedule adjustments | To ensure educational and clinical objectives are met post-parental leave. | Do you anticipate needing adjustments to your schedule? |
| Available mentorship programs | To provide guidance and advocacy for parental residents. | Would you like to be paired with a mentor experienced in managing parenthood? |
| Policies for workload distribution | To promote equitable workload during pregnancy and post-parental leave. | What specific barriers do you foresee in balancing residency and parenting? |
| Childcare support options | To reduce logistical barriers for resident parents. | Are you aware of the childcare options available through our institution? |
| Health and safety policies (e.g., radiation exposure) | To protect resident health during pregnancy. | Do you feel informed about health and safety protocols during pregnancy? |
| Communication channels | To clarify expectations and resources available for parental residents. | Is there any additional support you feel would benefit you? |
| Training flexibility | To ensure training remains flexible and comprehensive. | How can we adjust your training to accommodate family planning needs? |
